# Detecting pathogenic structural variation in families with undiagnosed rare disease in a national genome project

**DOI:** 10.1038/s41431-026-02210-x

**Published:** 2026-08-05

**Authors:** Prasun Dutta, Alistair T. Pagnamenta, Christelle Robert, Anthony E. F. McGuigan, Alison Ross, Edward S. Tobias, Ruth McGowan, Morad Ansari, David Baty, Jonathan Berg, Therese Bradley, Vera Cerqueira, Austin Diamond, Mihail Halachev, Anne Lampe, Alison Meynert, Caitlin Newman, Marian Thomson, Urmi Trivedi, Nicola Williams, Jing Yu, Javier Santoyo-Lopez, Zosia Miedzybrodzka, Jenny C. Taylor, Timothy J. Aitman

**Affiliations:** 1 Centre for Genomic and Experimental Medicine, MRC Institute of Genetics and Cancer, University of Edinburgh, Edinburgh, UK; 2 Centre for Human Genetics and NIHR Oxford Biomedical Research Centre, University of Oxford, Oxford, Oxfordshire UK; 3 North of Scotland Medical Genetic Service, NHS Grampian, Polwarth Building, Foresterhill, Aberdeen, UK; 4 West of Scotland Centre for Genomic Medicine, NHS Greater Glasgow & Clyde and University of Glasgow, Queen Elizabeth University Hospital, Glasgow, Scotland UK; 5 West of Scotland Centre for Genomic Medicine, Queen Elizabeth University Hospital, Glasgow, Scotland UK; 6 South East of Scotland Genetics Service, Western General Hospital, Edinburgh, UK; 7 East of Scotland Regional Genetics Service, NHS Tayside, Ninewells Hospital, Dundee, UK; 8 Laboratory Genetics, West of Scotland Centre for Genomic Medicine, Queen Elizabeth University Hospital, Glasgow, Scotland UK; 9 Bioinformatics Analysis Core, MRC Institute of Genetics and Cancer, University of Edinburgh, Edinburgh, UK; 10 Edinburgh Genomics, University of Edinburgh, Edinburgh, UK; 11 School of Medicine, Medical Sciences, Nutrition and Dentistry, University of Aberdeen, Aberdeen, UK; 12 Present Address: Institute of Biomedical and Clinical Science, University of Exeter Medical School, Royal Devon University Healthcare NHS Foundation Trust, Exeter, Devon UK; 13 Present Address: Sequoia Genetics, I-HUB, 84 Wood Ln, London, UK

**Keywords:** Medical genomics, Data processing

## Abstract

Whole-genome sequencing (WGS) projects for rare disease diagnosis typically yield a diagnostic rate of 25–41%, depending on the methods for patient selection and the extent of prior genetic testing. The Scottish Genomes Partnership (SGP) is a collaborative programme using genome sequencing to diagnose rare disease patients with presumed monogenic aetiology in the Scottish NHS. Within SGP, short-read sequencing (SRS) had previously achieved a diagnostic rate of 23% in affected families. To increase diagnostic yield, we applied Oxford Nanopore Technologies (ONT) long-read sequencing (LRS) to a cohort of 24 SGP families (74 individuals) that remained undiagnosed after SRS-based SNV/indel analysis. We also retrospectively reviewed SRS-derived structural variant (SV) calls to assess whether LRS results could have been detected in SRS data. After quality, rarity, panel-based and inheritance-based filtering, 392 candidate SVs were retained across de novo, homozygous recessive, compound heterozygous and X-linked inheritance models, of which 8 overlapped PanelApp diagnostic-grade “green” genes. Pathogenic or likely pathogenic de novo SVs were identified in 3 of 24 families: an *AUTS2* inversion, a *DLX5/6* locus inversion, and an *FN1* deletion. All three SVs were independently confirmed by retrospective SRS re-analysis using SVRare. These findings demonstrate that LRS-based SV analysis, supported by orthogonal SRS re-analysis, can resolve clinically significant SVs in families who remain unsolved after standard rare disease testing.

## Introduction

Rare diseases affect fewer than 1 in 2000 people in a population [1,2]. Up to 10,000 rare diseases [1] collectively affect 3–6% of the population worldwide [2]. Many individuals remain undiagnosed due to non-specific clinical presentation, resulting in prolonged diagnostic odysseys [3]. Early diagnosis can improve clinical management, guide access to targeted therapies, and reduce the prolonged uncertainty [4].

Genome sequencing in large, rare disease cohorts yields diagnoses in 25–41% of cases [5–9]. Traditional investigations have largely been superseded by WES/WGS [10–14], though most diagnoses arise from SNVs or small indels, with SVs less frequently implicated in diagnoses [6,8,15,16].

SVs are ≥50 bp genomic alterations including insertions, deletions, duplications, inversions and translocations that can cause monogenic Mendelian and complex diseases [17,18]. They have historically been detected by cytogenetic methods and, more recently, inferred from short-read sequencing (SRS) [19,20]. However, SRS suffers from mapping ambiguity in low-complexity regions, reducing sensitivity for SV detection [21,22].

Long-read sequencing (LRS) technologies, such as Oxford Nanopore Technologies (ONT) and PacBio, generate reads spanning kilobases to megabases [22,23]. These technologies can improve mapping across repetitive and difficult-to-map, low complexity regions, enabling detection of large SVs that are often missed by SRS [21,24–27]. Pathogenic SVs may therefore remain undetected in cases analysed only by SRS [28–30]. LRS also enables accurate short tandem repeat (STR) expansion detection, which contributes to a range of rare diseases, by spanning the full repeat and enabling direct resolution of repeat size and sequence composition in a single experiment [31]. Despite ongoing challenges, LRS increasingly identifies pathogenic SVs and improves diagnostic yield [28,32,33].

The Scottish Genomes Partnership (SGP) [8] is aligned with the 100,000 Genomes Project [6], with the aim of diagnosing Scottish patients with rare Mendelian disorders via in silico gene panel-based analysis of SRS data. Previous SGP SRS analysis of 999 genomes achieved a 23% diagnostic yield [8]. We applied LRS to 24 previously unsolved families to identify pathogenic SVs and improve diagnostic yield.

## Subjects and methods

### Patient recruitment and sample collection

Twenty-four families (74 individuals) recruited for the SGP LRS study were selected from those that did not receive a genetic diagnosis in the SGP SRS study [8]. Only families for whom samples were available from one or more affected probands and both unaffected parents (primarily trios or quads) were selected, thereby supporting the identification of de novo (autosomal dominant), recessive and X-linked variants. Selected families were prioritised based on a high clinical suspicion of a monogenic aetiology, indicated either by a shared phenotype among multiple family members, or a syndromic presentation suggestive of an underlying monogenic disorder.

All families had already undergone Illumina whole-genome SRS within the SGP SRS study [8]. This initial analysis was done in two stages and focused on the identification of only SNVs/indels, without systematic assessment of SVs as part of the clinical pipeline at that time. This initial panel-based review examined Tier 1 and Tier 2 variants (variants in PanelApp diagnostic-grade “green” genes), followed by an assessment of Tier 3 variants (predicted coding variants outside phenotype-matched panels) for trios. Only families who remained undiagnosed after both analytical stages were included in this LRS SV study and subsequent retrospective SRS SV re-analysis.

The 24 families in the LRS cohort were selected from the SGP SRS cohort. These families had already undergone both panel-based and broader protein-coding exome SNV/indel analysis but remained undiagnosed and were considered suitable for SV-focused analysis. In silico panel-based prioritisation of SVs was used to achieve a clinically practical workflow.

### DNA sample processing and sequencing

DNA extraction and sample processing were performed as previously described [8]. For inclusion, a minimum of 15 µg of genomic DNA, preferably with a DNA integrity number (DIN) > 8, was targeted from all family members. DNA shearing and library preparation are described in Supplementary Methods. Before sequencing the full cohort of 22 trios and 2 quads (*n* = 74), a pilot study using 7 samples from 4 patients was used to evaluate DNA quality and size selection and the suitability of clinical samples not originally collected for LRS. Sequencing was carried out at Edinburgh Genomics using ONT ligation kits (SQK-LSK109/114) on PromethION platforms with R9.4.1 and R10.4.1 flow cells (Supplementary Table 1).

### SV analysis

#### SV analysis and filtering

**Fig. 1:**
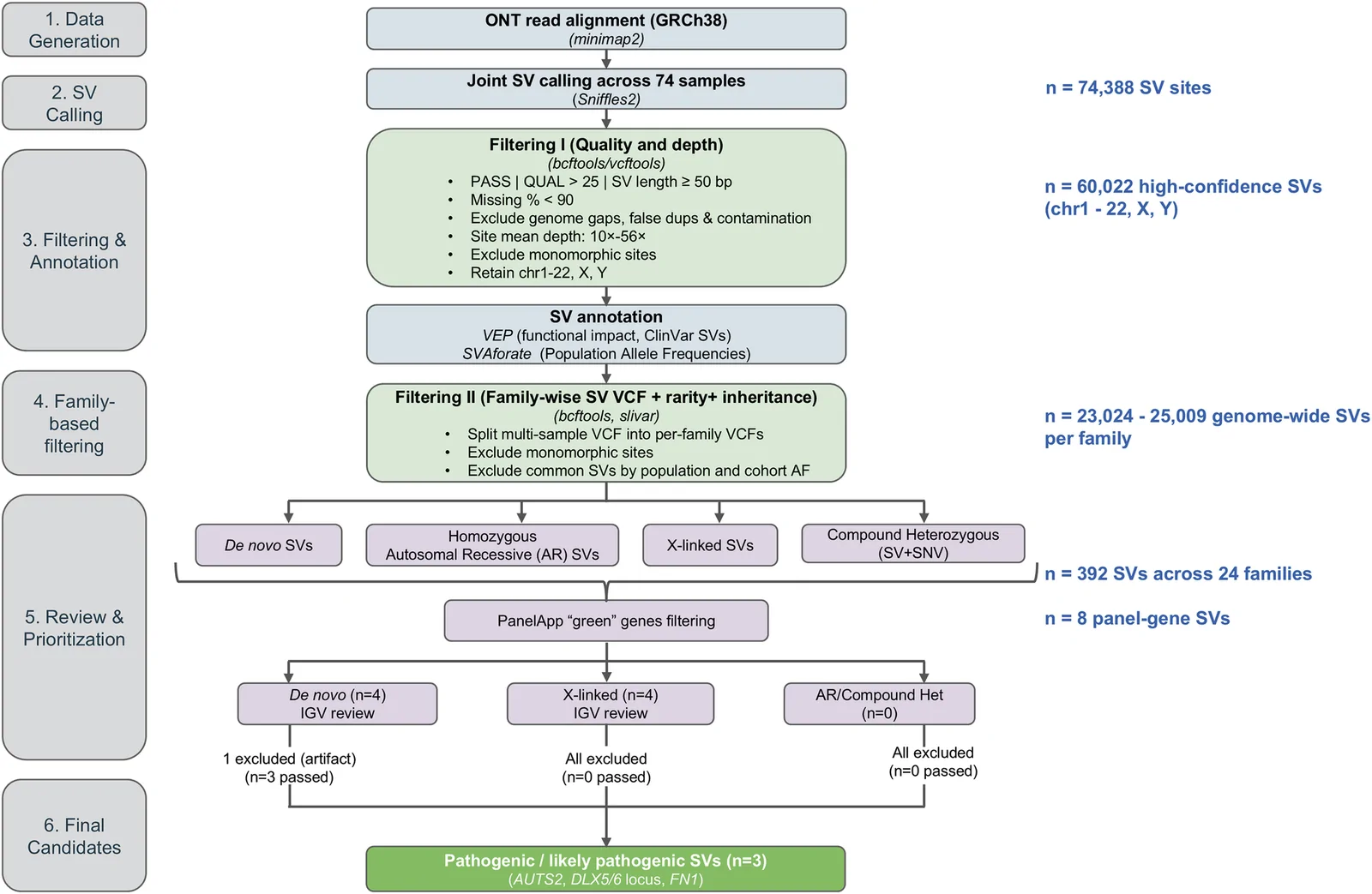
Schematic of the SV discovery and prioritisation workflow using LRS. Blue-grey boxes represent data generation and annotation steps. Green boxes represent quality and depth filtering steps applied at the cohort level. Purple boxes represent family-based inheritance filtering, clinical prioritisation via clinician-assigned PanelApp panels containing green (diagnostic-grade) genes, and manual IGV review steps. The bold green box indicates the final diagnostic outcome. SV counts (*n*=) at each stage are shown in blue. Four modes of inheritance were evaluated - de novo, homozygous autosomal recessive, X-linked and compound heterozygous (SV + SNV), reflecting the assumption that all parents were unaffected. Software tools are shown in italics. Three pathogenic or likely pathogenic SVs were retained as candidate diagnoses: in *AUTS2*, at the *DLX5/6* locus, and in *FN1*.

For the pilot study (*n* = 7), reads from PromethION Beta were base-called and demultiplexed with Guppy v4.0.11+f1071ce. “pass” tagged reads were filtered (<500 bp, quality score <9) and trimmed (40 bp start, 20 bp end) using NanoFilt v2.8.0, then aligned to GRCh38 using minimap2 v2.23 [34]. SVs were called with cuteSV v1.0.13 [35]. In the family-based study (*n* = 74), 73 samples were initially sequenced on an R9.4.1 flow cell (PromethION Beta). Among these, 28 were additionally sequenced on an R10.4.1 flow cell (PromethION 24) to increase coverage. One additional sample was sequenced only on R10.4.1, giving a total of 29 samples with R10.4.1 data. Base-calling and demultiplexing used Guppy v5.1.13 + b292f4d (PromethION Beta) and Guppy v6.5.7+ca6d6af (PromethION 24). “pass” tagged reads were filtered (<1000 bp, quality score <9) and trimmed (40 bp start, 20 bp end) using NanoFilt v2.8.0, then aligned to GRCh38 using minimap2 v2.23. Multi-sample SV calling was performed across all SGP LRS samples using Sniffles v2.0.6 [36]. Tools were selected based on benchmarking against GIAB reference data (see Supplementary Methods). Filtering and annotation of SVs was carried out as described in the Supplementary Methods, using standard and cohort-specific quality filters to remove artefacts, excluding common variants based on population databases and incorporating clinical and functional annotations to support downstream interpretation. A schematic diagram of the LRS SV calling and filtering workflow is shown in Fig. 1.

#### SV prioritisation

Analysis was initially restricted to clinician-assigned gene panels based on HPO terms. The panels were downloaded from PanelApp [37] with analysis limited to diagnostic-grade “green” genes. SVs were then evaluated under four modes of inheritance (MOI) consistent with unaffected parents using slivar v0.3.1 [38]: de novo autosomal dominant, homozygous autosomal recessive, compound heterozygous (SV + SNV/small indel from SRS data) and X-linked (in male probands). Variants retained after technical review and phenotype/inheritance prioritisation were discussed at MDT meetings. More detailed variant prioritisation rules are provided in Supplementary Methods.

A secondary analysis was carried out to explore variants beyond pre-defined gene panels. Genome-wide putative de novo SVs annotated by VEP [39] with HIGH or MODERATE impact were reviewed without restriction to gene panels and a genome-wide compound heterozygous analysis was done by intersecting SVs with SNVs in OMIM-annotated biallelic disease genes, with filters applied for rarity, quality, VEP impact (HIGH or MODERATE) and phase (in trans).

#### SRS SV analysis using SVRare

Methods for genome sequencing and data processing of the SGP SRS samples in the 100,000 Genomes Project have been described previously [8]. Briefly, 150 bp paired-end Illumina HiSeqX reads were aligned to GRCh37/38 via iSAAC aligner [40], with SVs called via Manta [41] and Canvas [42]. However, the SV calls were not analysed as part of the original GEL pipeline. The SRS SV calls were only subsequently processed, once LRS had been undertaken, using SVRare, a genome-wide prioritisation framework that aggregates SV and CNV calls from multiple callers to identify rare, clinically significant variants [43]. SVRare filters candidates based on cohort-wide allele frequency (AF) and prioritises those affecting genes linked to participant HPO terms or predefined gene panels. SVRare was not available at the time of the initial SV analysis but was used here to retrospectively collate SVs from SRS data and provide orthogonal confirmation of LRS-derived SVs.

## Results

### Pilot study: effects of DNA quality and size selection on ONT sequencing and SV detection

To assess the effects of DNA quality, size selection and fragmentation on SV discovery, seven samples from four patients were sequenced on an R9.4.1 flow cell. Most samples achieved 6–11× median depth of coverage with yield correlating with median/mean coverage but not with read count (Table 1). Size selection had little effect on sequence coverage but substantially improved read length profiles, with most reads in size-selected samples exceeding 10 kb and some reaching 80–100 kb. A partially degraded sample, Sample 4 (size-selected), performed well and achieved high coverage, suggesting that size selection can enable successful LRS from stored DNA samples not specifically prepared for LRS (Supplementary Fig. 1A).

**Table 1:** Sequencing and alignment statistics for the 7 pilot samples.

Sample Name	Size selected?	Yield (bp)	Median read length (bp)	Read length N50 (bp)	Aligned reads	Median coverage	Mean coverage
1a	N	20,17,85,37,964	7494	14,459	21,07,646	6.0×	6.8×
1b	Y	31,80,88,32,054	14,074	17,001	21,87,913	10.0×	10.5×
2a	N	34,14,55,43,065	8739	15,046	31,99,074	11.0×	11.5×
2b	Y	34,86,32,71,406	13,751	17,757	24,02,842	11.0×	11.5×
3a	N	32,20,25,47,854	10,037	18,305	26,09,081	10.0×	10.9×
3b	Y	25,32,45,02,117	17,185	22,736	14,29,244	8.0×	8.4×
4	Y (degraded)	34,38,42,35,565	13,233	18,502	24,88,596	11.0×	11.4×

Samples labelled ‘a’ and ‘b’ are non-size-selected and size-selected libraries from the same individual, respectively. Sample 4 was partially degraded. Only primary alignments are counted. Coverage is reported against GRCh38. Statistics calculated using Qualimap and NanoComp.

Between 10,000 and 25,000 putative SVs were detected across the 7 samples with greater sequencing depth yielding higher SV counts (Supplementary Fig. 1B). SV type distribution was consistent with previous studies [44].

After retaining SVs ≥50 bp, the union set contained ~32,000 SVs, with lengths >80 kb (Supplementary Fig. 1C). Peaks of SVs were observed at around 300 bp and 6000 bp corresponding to detection of *Alu* and LINE-1 elements, consistent with previous reports [45] (Supplementary Fig. 1C). Overall, this pilot demonstrated that clinical DNA samples, including partially degraded DNA, can be used for reliable SV detection using LRS.

### Sequencing results of family samples (*n* = 74)

Per sample read counts and QC statistics for R9.4.1 and R10.4.1 sequencing runs are provided in Supplementary Tables 2–5. Key QC metrics before and after trimming and filtering are shown in Supplementary Fig. 2. Filtering improved median read length, quality and N50, and R10.4.1 flow cells showed higher and more consistent read quality than R9.4.1 (Supplementary Fig. 2A–D).

Read alignment to GRCh38 resulted in a mean depth of coverage per sample ranging from 10× to 57×, with >99% mapping rate and 91.5–97.2% identity (Supplementary Table 6, Supplementary Fig. 3A). Coverage from R10.4.1 flow cells was noticeably higher than from R9.4.1 flow cells (Supplementary Fig. 3B).

### SV-calling statistics in SGP LRS families

**Fig. 2:**
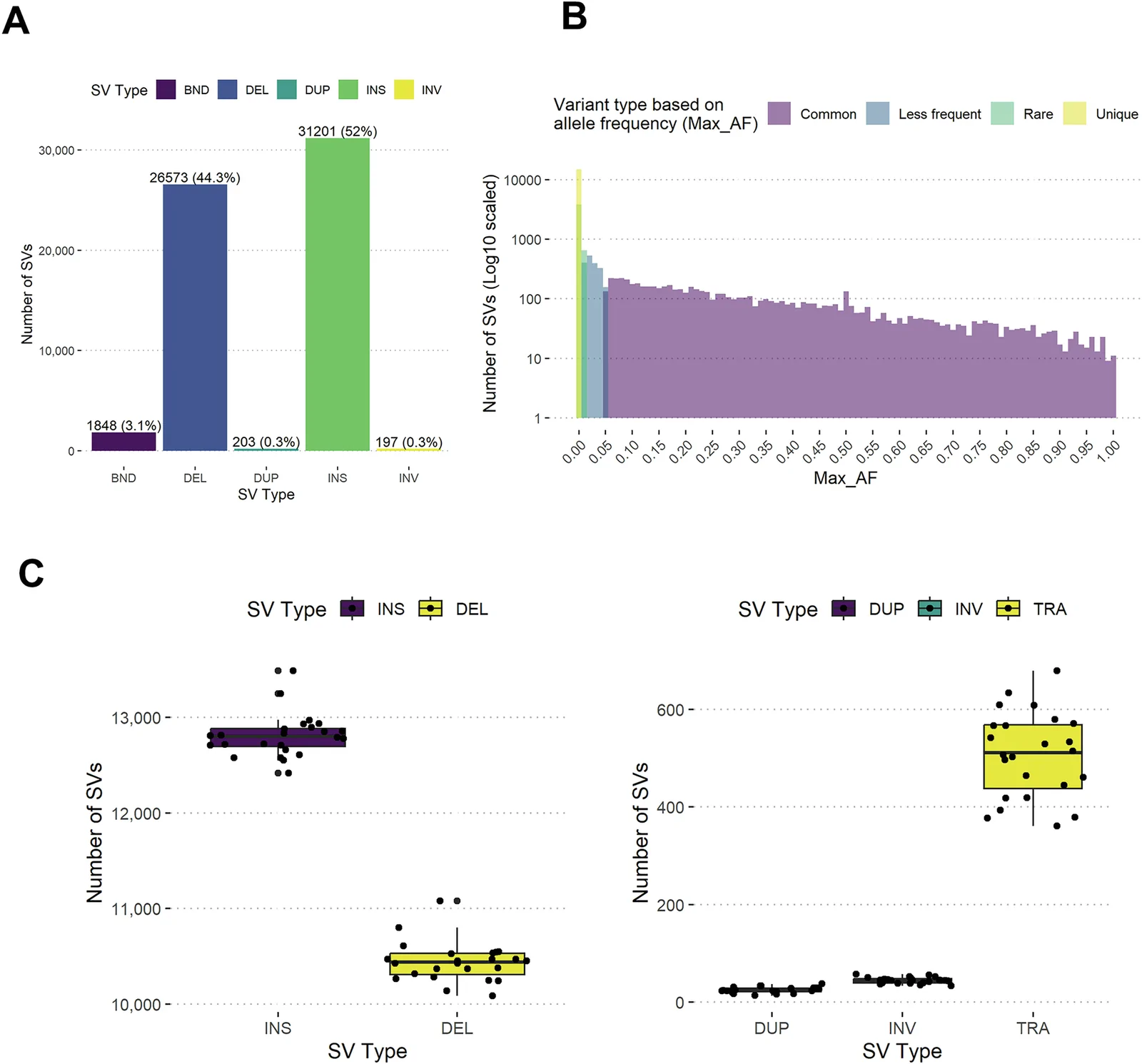
SV Distribution by Type and Frequency. **A** Barplot showing the number of filtered SVs stratified by SV type. **B** Histogram showing the number of filtered SVs that are common (Max_AF or maximum allele frequency ≥ 0.05), less frequent (Max_AF < 0.05 and ≥ 0.01), rare (Max_AF < 0.01 and Max_AF ≠ 0) and unique to the SGP LRS cohort (Max_AF = 0). **C** Box and jitter plot showing the distribution of the number of filtered INSs, DELs, DUPs, INVs and TRAs per family (*n* = 24).

Multi-sample SV-calling identified 74,388 putative SVs across 74 individuals, representing all variants relative to the GRCh38 reference genome prior to filtering. To minimise artefactual calls from low or unusually high depth, the distribution of site mean read depth (mean depth per SV site averaged across individuals) was examined (Supplementary Fig. 4). A depth filter of 10–56× was applied based on this empirical distribution, removing SVs in low-coverage regions or those with inflated depth likely reflecting alignment artefacts or repetitive sequence. The upper threshold (56×) corresponds to approximately twice the mean site-level depth. After applying additional quality filters (see Methods and Supplementary Methods), 60,022 high-confidence SVs were retained across chromosomes 1–22, X and Y. Most SVs were insertions (52%) and deletions (~44%), followed by translocations (~3%), duplications (0.3%) and inversions (0.3%) (Fig. 2A). Most SVs (61.9%) were unique to the SGP LRS cohort (Max_AF = 0), with no matches in SVAFotate databases (Supplementary Fig. 5). Most SVs were either unique to the cohort or rare, as reflected in the absolute counts across frequency categories (Fig. 2B). The percentage distribution of common, less frequent, rare and unique SVs was similar across SV types, except for duplications, where rare SVs were more abundant (Supplementary Fig. 6). SV counts per family did not show any major outliers (Fig. 2C, Supplementary Table 7).

### SV prioritisation and analytical strategy

An SV prioritisation pipeline was devised to reduce the calls to a manageable number of candidate SVs for manual review (Fig. 1). The cohort-level SV call set was converted to family-specific datasets, with each family retaining 23,024–25,009 genome-wide SVs (Supplementary Table 7). After quality filtering, and functional annotation with VEP, SVs were prioritised within clinician-assigned, family-specific PanelApp gene panels. Rarity and inheritance-based filtering yielded 392 candidate SVs across all families (Table 2, columns D-K, summed across families). This primary analysis reflects a clinically driven, panel-based prioritisation strategy.

**Table 2:** Summary of SVs per family.

			De novo		X-linked		Autosomal recessive (homozygous)		Compound heterozygous	
S.No.	Family_ID	Total_SVs	Genome-wide	Panel genes	Genome-wide	Panel genes	Genome-wide	Panel genes	Genome-wide	Panel genes
1	Family_1	23,024	2	1	6	0	4	0	1	0
2	Family_2	23,527	8	0	0	0	4	0	0	0
3	Family_3	23,403	3	1	4	0	0	0	9	0
4	Family_4	24,276	2	0	0	0	0	0	0	0
5	Family_5	23,869	10	0	1	0	1	0	0	0
6	Family_6	23,809	7	0	2	0	2	0	4	0
7	Family_7	23,930	5	0	2	0	1	0	1	0
8	Family_8	24,001	1	0	4	1	2	0	0	0
9	Family_9	23,655	3	0	0	0	1	0	3	0
10	Family_10	23,633	2	0	0	0	3	0	2	0
11	Family_11	23,374	5	1	0	0	0	0	2	0
12	Family_12	24,656	13	0	0	0	0	0	5	0
13	Family_13	23,499	4	0	0	0	5	0	0	0
14	Family_14	25,009	7	0	5	0	0	0	29	0
15	Family_15	23,650	4	0	0	0	0	0	0	0
16	Family_16	24,012	36	1	0	0	3	0	1	0
17	Family_17	23,797	3	0	5	1	5	0	0	0
18	Family_18	23,273	2	0	5	2	4	0	7	0
19	Family_19	23,863	9	0	0	0	0	0	1	0
20	Family_20	23,819	14	0	0	0	1	0	1	0
21	Family_21	23,917	17	0	4	0	2	0	1	0
22	Family_22	24,001	14	0	5	0	1	0	1	0
23	Family_23	24,041	46	0	0	0	3	0	1	0
24	Family_24	23,609	12	0	5	0	3	0	1	0

SV counts per family stratified by mode of inheritance, genome-wide and within clinician-assigned disease gene panels comprising diagnostic-grade PanelApp “green” genes. Total_SVs represents family-specific SVs retained after quality and depth filtering. Counts in columns D-K represent a subset of these SVs after additional filtering based on various mode of inheritance (MOI). The total number of MOI-prioritised SVs across all families is 392, of which 8 fell within panel genes classified as “green” in patient-specific, clinician-assigned panels.

Within this set, the number of genome-wide de novo SVs identified under the de novo inheritance model was higher than expected (up to 46 in a single family; Table 2, “De Novo: Genome-wide” column). Each generation is expected to have at most one true de novo SV [18]. This inflation is likely due to missed detection of variants in parents at lower coverage, leading to inherited variants being misclassified as de novo. Families sequenced using both R9.4.1 and R10.4.1 flow cells showed fewer such calls than those sequenced only on R9.4.1 (Supplementary Fig. 7). These calls were reduced through downstream filtering and manual curation.

Of the 392 candidate SVs, 8 SVs overlapped diagnostic-grade “green” genes in PanelApp gene panels before manual curation, comprising 4 de novo and 4 X-linked candidates. These candidates were reviewed in IGV [46] to exclude artefactual SVs. Following this review, one de novo SV was excluded, while the remaining three de novo SVs, corresponding to the three exemplar families described below, were prioritised for further evaluation. These were then discussed with referring clinicians at MDT meetings to confirm genotype-phenotype concordance. All X-linked candidates were excluded because of inconsistent segregation patterns or insufficient evidence for functional or regulatory relevance.

Genome-wide examination of X-linked SVs did not identify additional candidate diagnoses, as most variants were intergenic or intronic, or showed phenotype mismatch, although long-range regulatory effects cannot be excluded.

No SVs overlapping biallelic panel genes with autosomal homozygous recessive MOI were identified in this cohort. Additionally, no compound heterozygous scenarios (SV+SNVs) were found in biallelic panel genes for any family.

A secondary genome-wide analysis, independent of PanelApp panels, was also undertaken using a distinct filtering strategy focused on de novo SVs with predicted functional impact. In the first phase, genome-wide putative de novo SVs annotated by VEP as HIGH or MODERATE impact were investigated. This yielded 66 unique variants across 20 families (Supplementary Table 8), which were manually reviewed in IGV. Many SVs occurred in low-complexity or repetitive regions and showed alignment patterns suggestive of artefacts. Several putative de novo SVs were reclassified as inherited or present in all three family members, indicating false-positive calls. A subset of events affected lncRNAs, non-coding transcripts, or pseudogenes, limiting clinical relevance. Manual review substantially reduced the candidate set to 10 variants, labelled as “Retained” in Supplementary Table 8. However, none of the retained variants overlapped OMIM genes with established disease associations.

In the secondary genome-wide analysis, SV + SNV combinations in OMIM-annotated biallelic disease genes were evaluated, but all were excluded after curation because in each case at least one variant was benign (Supplementary Table 9).

A compilation of genome-wide de novo SVs and compound heterozygous instances (SV+SNVs) is shown in Supplementary Tables 10 and 11, respectively. These are provided for future gene discovery and investigation of SV regulatory effects.

### Families with SVs in PanelApp genes

Pathogenic or likely pathogenic SVs were identified in three exemplar families. IGV plots generated from the LRS data are shown in Supplementary Fig. 8. SVs in Families 1 and 2 were accepted as pathogenic at MDT review meetings while Family 3 SV was designated as likely pathogenic.

### Family 1 (recruited disease: intellectual disability)

The proband is a male and the firstborn child of nonconsanguineous and unaffected parents. He was referred to Clinical Genetics at the age of 6.9 years for an undiagnosed neurodevelopmental disorder and recent onset of seizures. His medical issues included unclassified epilepsy, developmental delay (nonverbal), microcephaly (−3.5 s.d.), sleep disturbance, skeletal abnormalities (finger contractures, bilateral calcaneal valgus with foot pain) and convergent squint. Examination showed microcephaly with flattened occiput, convergent squint, mid-face hypoplasia, micrognathia, prominent nose/flattened nasal bridge, short philtrum, prominent ears, thickened lips and joint contractures.

Brain MRI (age 8), electroencephalogram (EEG) (age 9) and metabolic screening were normal. Chromosome microarray and exome sequencing through Deciphering Developmental Disorders [15] (DDD) study were non-diagnostic. He was subsequently recruited to SGP, where SRS analysis did not identify any Tier 1, 2, or 3 variants.

**Fig. 3:**
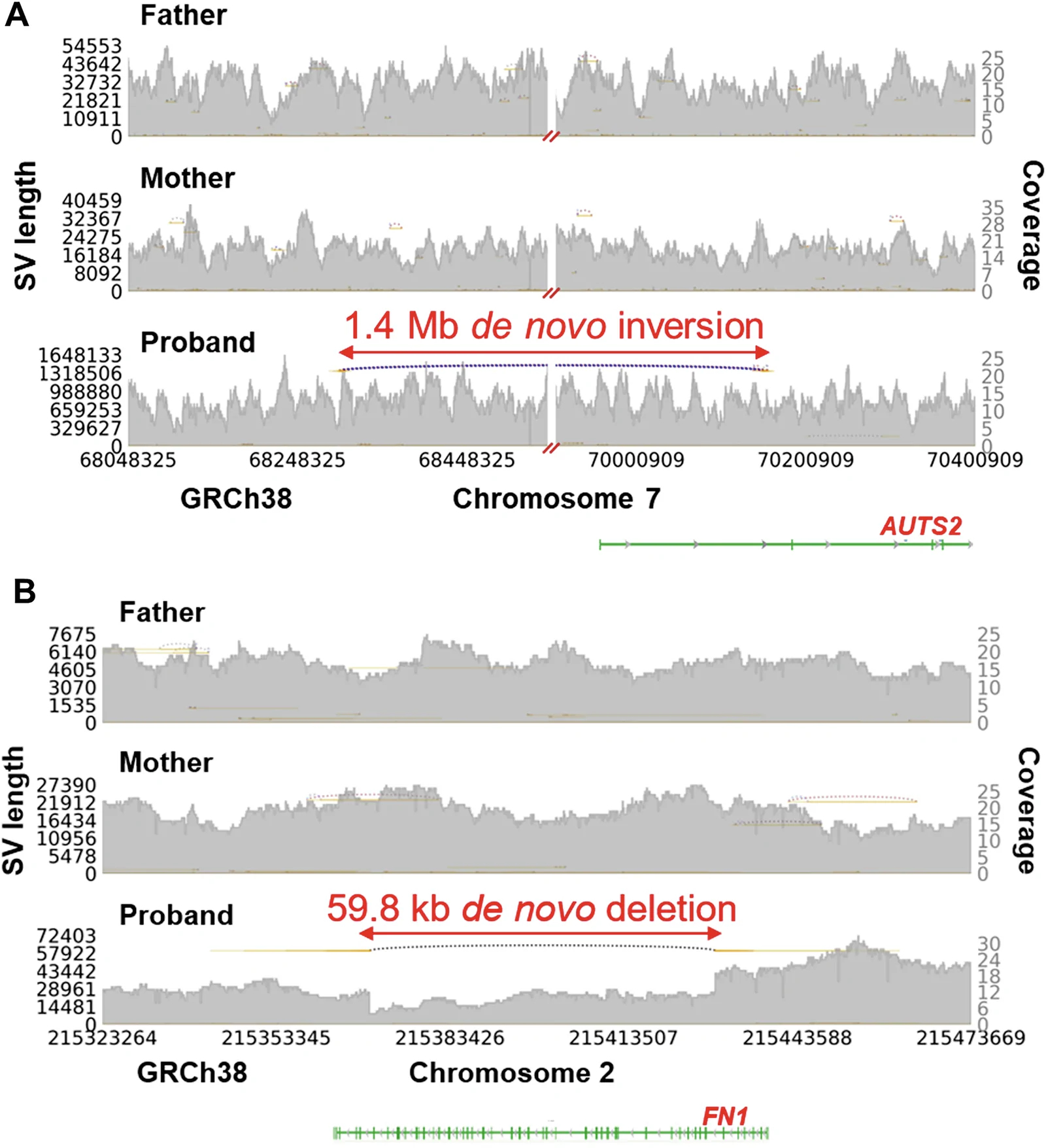
Exemplar De Novo SVs. **A** A 1.4 Mb de novo inversion found from LRS data in the proband from exemplar family 1 that overlaps with *AUTS2* and is absent in the parents. The inversion is denoted by a purple dotted line in the proband. Other smaller SVs can be ignored. The x-axis refers to the chromosome base position. The left y-axis refers to the SV length and the right y-axis represents the depth of coverage. **B** A 59.8 kb de novo deletion found from LRS data in the proband from exemplar family 3. The multi-exon deletion is found within *FN1*, with exons 6 to 41 of the MANE transcript deleted.

LRS detected a 1.4 Mb de novo inversion disrupting *AUTS2* (MIM: 615834), a candidate gene for neurodevelopmental and neurological disorders encoding the Activator of Transcription and Developmental Regulator protein [47], which mediates neurogenesis, neuronal maturation, synapse formation and dendritic spine regulation [48]. One inversion breakpoint was located within intron 2 and was predicted to have a high functional impact on the protein-coding sequence, removing exons 1 and 2. This disruption is anticipated to impair gene expression and is consistent with the proband’s phenotype. This inversion was also detectable on retrospective analysis of the SRS data and was prioritised using SVRare, providing orthogonal support for the event [49]. A samplot [50] image of the LRS inversion is shown in Fig. 3A and an IGV plot is shown in Supplementary Fig. 8A. The SV was classified as pathogenic according to ACMG guidelines.

### Family 2 (recruited disease: ultra-rare undescribed monogenic disorders)

The proband is a female with three-limb ectrodactyly (both hands and right foot), retro-micrognathia with dental overcrowding and overfolded ear helices. Audiometry revealed narrow ear canals and mild mixed hearing loss. Resting sinus bradycardia was noted with normal cardiac structure. Ectrodactyly was observed antenatally in the proband’s daughter. Additionally, patent ductus arteriosus, atrial septal defect and pulmonary valve stenosis were noted at birth. The proband’s parents are unaffected.

**Fig. 4:**
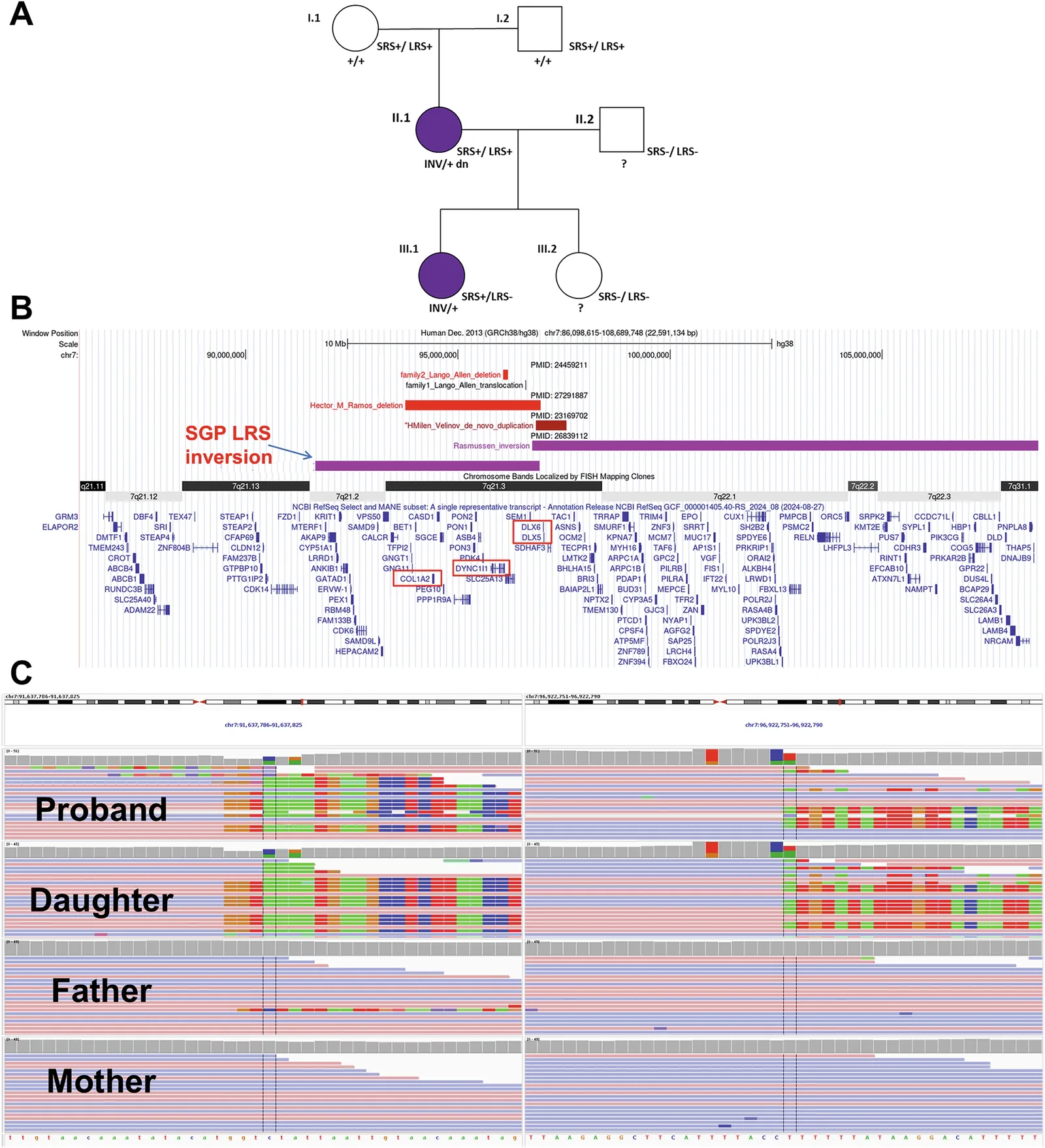
Inversion near DLX5/6 at the SHFM1 Locus. **A** Pedigree diagram showing the three generations of exemplar family 2. Sequence was unavailable for family members II.2 and III.2. SRS and LRS labels along with the symbols “+” or “-” indicate the availability and unavailability of the type of sequencing data, respectively. The proband (II.1) carries a 5.2 Mb heterozygous de novo inversion (denoted as “dn”) near the *DLX5/6* locus, which was inherited by her daughter (III.1). **B** The UCSC session displays the *SHFM1* locus (7q21) with previously reported SVs in various publications, alongside the inversion identified in the SGP LRS patient reads. **C** IGV plot showing soft-clipped bases near the inversion breakpoint in the proband and her daughter, which are absent in the parents of the proband. The IGV plot was generated using SRS data available in the Genomics England (GEL) research environment and includes an additional family member, the daughter of the proband, sequenced by SRS.

For this family, samples from the unaffected parents and proband underwent LRS. SRS was available for the trio and, additionally, the proband’s affected daughter. Trio LRS identified a 5.2 Mb de novo inversion in the split hand/foot malformation type 1 (*SHFM1*, MIM: 183600) region, previously reported in association with limb malformation (Fig. 4B) [51–54]. This event was prioritised because the panel gene *COL1A2* was present within the inversion, although neither breakpoint directly disrupted the gene. Closer inspection of the region showed that *DLX5* and *DLX6*, important genes in the WNT pathway involved in limb development [53], were located close to the distal breakpoint. *DYNC1I1*, located at one end of the inversion, contains enhancer elements that regulate *DLX5*/*6* and the inversion is predicted to reposition these enhancers away from their target genes. Disruption of these enhancer-gene interactions is likely to reduce *DLX5/6* expression, consistent with the split hand/foot phenotype [51–54]. SRS data from the proband’s affected daughter, who also has ectrodactyly, was found to carry the inversion when analysed retrospectively using SVRare (Fig. 4A, C), providing orthogonal confirmation of the inversion. IGV visualisation of the LRS data is shown in Supplementary Fig. 8B. The SV was classified as pathogenic according to ACMG guidelines.

### Family 3 (recruited disease: unexplained skeletal dysplasia)

The proband is a male and the only child of nonconsanguineous healthy Scottish parents. He presented at age 8 with abnormal gait and bilateral hip pain (onset at 6–7.5 years) and was regarded as bilateral Perthes-like disease. Progressive contractures developed in the hips from age 12 and elbows from 14, along with pain and swelling of interphalangeal joints from age 14, requiring intra-articular injections.

On examination, there was reduced hip movement and significant girdle weakness with normal muscle power and reflexes. He walked with thoracic kyphosis, lumbar lordosis, flexed knees and stiff hips. His height, proportion, vision, hearing and teeth were normal and he was non-dysmorphic. A skeletal survey (age 11.6 years) showed slightly ovoid vertebral bodies. Bone age was appropriate. Pelvic MRI revealed pelvic muscle atrophy without evidence of myopathy. Hip MRI revealed fragmented, collapsed, irregular proximal femoral epiphyses. Further review revealed bilateral symmetrical irregular sclerosis of the femoral epiphyses, mild metaphyseal broadening with minor cystic changes and subchondral fracturing and collapse, in addition to bilateral hip joint effusions and lumbar spine end-plate concavities. Muscle biopsy with ultrastructural analysis was unhelpful and serum creatine kinase, nerve conduction, electromyograms and lumbar punctures were normal. A diagnosis of multiple epiphyseal dysplasia was considered.

Sanger DNA sequencing of 5 MED-related genes (*COMP, COL9A1, COL9A2, COL9A3 and MATN3*), in addition to *LMNA*, had been performed, but no pathogenic variant was identified. Chromosomal microarray revealed a de novo heterozygous deletion (~55 kb) at *2q35* in the *FN1* gene (MIM: 184255), involving multiple exons which was classified as a VUS. Additionally, the SGP SRS study utilised gene panels for various skeletal and neuromuscular disorders, but initial analysis found no causative variants. The deletion was likely present in the SRS data but was not prioritised due to the absence of systematic SV review in the clinical pipeline at that time.

LRS identified a 59.8 kb de novo multi-exon in-frame deletion in *FN1*, spanning exons 6–41 (out of a total of 46 exons in the MANE Select transcript) (Fig. 3B, Supplementary Fig. 8C), confirming the chromosomal microarray result and precisely defining the deletion breakpoints that the array could not provide. IGV visualisation showed a clear drop in the depth of coverage in the proband compared with both parents. The deletion was also identified in retrospective SVRare analysis of the SRS data. In the musculoskeletal system, *FN1* encodes an extracellular-matrix multimeric glycoprotein involved in cell adhesion and migration [55]. The *FN1* deletion was initially mis-genotyped as homozygous by the LRS SV caller, leading to its exclusion under a strict cohort AF threshold of 0.01. Visual inspection of read alignments clarified the heterozygous state, highlighting the sensitivity of variant prioritisation to genotyping errors and AF thresholds. Using a more lenient threshold of 0.015 retained the variant. The *FN1* SV was reclassified as likely pathogenic according to ACMG guidelines and additional work is ongoing to establish a definitive connection between *FN1* and bilateral Perthes-like disease.

A comparison of SV detection across the original SGP SRS analysis, LRS analysis and retrospective SRS re-analysis is summarised in Table 3.

**Table 3:** SV detection comparison across original SGP SRS, LRS, and retrospective SRS SV analysis (SVRare) for the three exemplar families.

Exemplar Family	SV type	Gene/locus	Original SGP SRS analysis	LRS analysis	Retrospective SRS SV analysis (SVRare)	Interpretation
1	Inversion (1.4 Mb)	*AUTS2*	Not reported in initial panel-based analysis	Detected	Detected	Not prioritised in the initial SGP SRS analysis (no SV-focused review)
2	Inversion (5.2 Mb)	*DLX5/6* locus (SHFM1 region)	Not reported in initial analysis	Detected	Detected but not initially prioritised	Not prioritised in SRS panel-based analysis (regulatory/positional effects not captured)
3	Deletion (59.8 kb)	*FN1*	Detected by microarray (VUS); not prioritised in SRS analysis	Detected	Detected	Present in SRS but not prioritised; microarray lacked breakpoint resolution

For each family, the SV type, affected gene or locus, and detection status across the three analytical approaches are shown. The Interpretation column summarises the reason why each SV was not identified or prioritised in the original SGP SRS analysis.

## Discussion

LRS, supported by orthogonal SRS-based SV analysis, enabled a diagnosis or likely diagnosis in 3 out of 24 families that had remained undiagnosed after prior extensive genetic testing. This was achieved using a panel-based strategy directly comparable to a standard clinical workflow, integrating automated SV calling with manual IGV review and MDT discussion across 74 individuals from 24 families.

Our pilot study examined how DNA quality and fragment size affect sequencing performance and SV discovery. Size-selection of partially degraded DNA produced comparable coverage and SV call profiles to those of high-quality samples and, although it increased the proportion of reads >10 kb, it did not significantly alter the SV calling rate. ONT protocols using R10.4.1 flow cells did not require DNA shearing at the time of this study, which is now recommended. A major difference was observed in sequence read depth and yield between the R9.4.1 and R10.4.1 flow cells, providing increased depth of coverage and more accurate SV calling and genotyping.

Distinguishing true pathogenic SVs from artefact and common variation was central to our analytical strategy. We first applied a combination of standard and cohort-specific quality filters (see Methods and Supplementary Methods) on genome-wide SV calls to remove artefacts and then utilised SV population databases to remove common SVs, while VEP, ClinVar and OMIM annotations were added to support downstream interpretation. SVs were prioritised using clinician-assigned PanelApp gene panels, and evaluated under de novo, recessive, compound heterozygous and X-linked models. Candidates were cross-validated with SVRare, reviewed in IGV and discussed at MDT meetings to confirm genotype-phenotype concordance.

These analytical decisions led to two pathogenic variant findings (*AUTS2 and DLX5/6 locus*) and one likely pathogenic finding (*FN1*). *AUTS2* is well established in a range of neurodevelopmental disorders, dysmorphic features and skeletal abnormalities, supporting its role in the phenotype of the Family 1 proband [48]. The novel *DLX5/6* inversion linked to ectrodactyly in Family 2 builds on literature defining critical regulatory regions for limb development, supporting a positional effect through disruption of enhancer-gene interactions at the *DLX5/6* locus [51–54]. For Family 3, the *FN1* deletion was initially rejected as radiological review did not show corner fractures typically associated with *FN1* mutations but was classified as likely pathogenic following MDT review given the close phenotypic match. Although most reported *FN1* variants are missense or splice-site changes, this de novo multi-exon deletion is predicted to cause loss-of-function. *FN1* is highly constrained against such variation (gnomAD v4.1.1 pLI = 1), supporting haploinsufficiency. No additional tiered variants in *FN1* were identified and further investigations are ongoing to clarify its involvement in the proband’s bilateral Perthes features.

While our primary analysis focused on coding SVs within panel genes to facilitate clinical interpretation, we also implemented a secondary genome-wide analysis of autosomal de novo SVs with predicted functional impact. However, this did not yield additional clinically relevant findings. Many SVs were filtered out following manual review, largely due to artefactual signals or localisation to repetitive or non-coding regions, highlighting the importance of stringent filtering and phenotype-driven prioritisation. In three examples, LRS provided improved alignment in repeat regions that were poorly resolved by SRS (Supplementary Table 8), highlighting the inherent technical limitations of SRS.

We acknowledge that this study has some limitations. Coverage was uneven across the cohort. Samples sequenced only on the R9.4.1 flow cell had lower depth and higher rates of genotyping error for de novo SVs and updating these to R10.4.1 could uncover additional diagnoses. Further analysis of genes outside the panels may yield additional diagnoses. We have catalogued the genome-wide de novo SVs and compound heterozygous (SV + SNV) events to support future gene discovery and studies of SV regulatory effects (Supplementary Tables 10 and 11, respectively). Our primary analysis was restricted to clinician-assigned PanelApp panels available at the time. Newer updates may include recently identified disease genes [37] and could yield additional diagnoses. We did not perform a genome-wide analysis of runs of homozygosity (ROH) or absence of heterozygosity (AOH), which identify regions of shared parental ancestry where recessive disease-causing variants are more likely to reside.

Finally, advances in SV calling algorithms, including new deep learning-based methods such as Cue [56], which leverage image representations of read alignments to improve SV detection and genotyping, may enable identification of additional pathogenic variants. However, many such methods do not yet support multi-sample or family-based analyses, which were essential for this study.

The exemplar cases described in this cohort demonstrate the potential of LRS identify SVs and uncover clinically relevant variants in cases that remain unsolved after standard analysis. Further studies are ongoing to establish the clinical relevance of genome-wide de novo SVs for selected families in this cohort.

Several studies have shown that LRS has resolved previously unsolved cases after SRS failed [28,33]. The SGP LRS cohort comprised 24 families deliberately selected from the original SGP study [8] that remained unsolved after extensive prior genetic testing, and LRS enabled a diagnosis or likely diagnosis in 3 of these families, with pathogenic or likely pathogenic SVs identified in cases where prior microarray, exome and genome-based SNV analysis had been non-diagnostic: an *AUTS2* inversion, an *FN1* deletion and a *DLX5/6* locus inversion. The ability to comprehensively identify SVs through LRS, in tandem with improving SV calling tools, creates new opportunities for molecular diagnoses in rare disease. Further analyses remain possible, but the exemplars demonstrate the added diagnostic value of LRS in rare disease diagnosis.

## Supplementary information

Supplementary Tables, Figures and Methods

Supplementary Table 1

Supplementary Table 2

Supplementary Table 3

Supplementary Table 6

Supplementary Table 8

Supplementary Table 9

Supplementary Table 10

Supplementary Table 11

## Data Availability

Data from the National Genomic Research Library (NGRL) used in this research are available within the secure Genomics England Research Environment. Access to NGRL data is restricted to adhere to consent requirements and protect participant privacy. Data used in this research include: ONT data: Aligned BAM files for 73 samples, family-specific SV VCFs for 24 families and a multi-sample SV VCF containing all 73 samples are available within the Genomics England Research Environment (GEL RE). One participant from the original cohort of 74 was removed for ethical reasons. File paths for single-sample and family-level BAM and VCF files are provided in the LabKey table “rare_disease_ont_sgp” within the project main-programme_v19_2024-10-31. The multi-sample SV VCF file can be found in the folder: /gel_data_resources/LRS_cohort_genomes/Rare_Disease/SGP2_LRS/Aggregate_VCF/. Further details about the dataset are available at: https://re-docs.genomicsengland.co.uk/rd_sgp_ont/. Illumina data: SRS data for participants was accessed through the LabKey table “rare_disease_analysis” within the project main-programme_v19_2024-10-31. Access to NGRL data is provided to approved researchers who are members of the Genomics England Research Network, subject to institutional access agreements and research project approval under participant-led governance. For more information on data access, visit: https://www.genomicsengland.co.uk/research. GRCh37 human reference genome and index: https://ftp-trace.ncbi.nlm.nih.gov/ReferenceSamples/giab/release/references/GRCh37/hs37d5.fa.gz. https://ftp-trace.ncbi.nlm.nih.gov/ReferenceSamples/giab/release/references/GRCh37/hs37d5.fa.gz.fai. GRCh38 human reference genome and index: ftp://ftp.ncbi.nlm.nih.gov/genomes/all/GCA/000/001/405/GCA_000001405.15_GRCh38/seqs_for_alignment_pipelines.ucsc_ids/GCA_000001405.15_GRCh38_no_alt_plus_hs38d1_analysis_set.fna.gz. ftp://ftp.ncbi.nlm.nih.gov/genomes/all/GCA/000/001/405/GCA_000001405.15_GRCh38/seqs_for_alignment_pipelines.ucsc_ids/GCA_000001405.15_GRCh38_no_alt_plus_hs38d1_analysis_set.fna.fai. PanelApp website: https://panelapp.genomicsengland.co.uk/.

## Code availability

The codes and custom scripts used in this manuscript will be made available upon request.
