# Supplementary material for: Detecting pathogenic structural variation in families with undiagnosed rare disease in a national genome project
Source: Eur J Hum Genet. 2026 Aug 5;34(10):1513–23. doi: 10.1038/s41431-026-02210-x (PMC13634035; doi:10.1038/s41431-026-02210-x)
Supplement: Supplementary file 1 [file 41431_2026_2210_MOESM1_ESM.pdf]

Detecting pathogenic structural variation in families with undiagnosed rare disease in a national genome project

Supplementary Tables, Figures and Methods

The Supplementary Information file consists of a single combined PDF containing Supplementary Figures 1-8, detailed methods, and Supplementary Tables 4, 5 and 7. Supplementary Tables 1, 2, 3, 6, 8, 9, 10 and 11 are provided as separate Excel files because they exceed one page in length, with their captions included in the PDF to preserve the table numbering.

Supplementary Tables

Supplementary Table 1: Family structure and flow cell type for all 74 sequenced samples.

Supplementary Table 2: Raw FASTQ QC metrics for 73 samples sequenced using R9.4.1 flow cell.

Supplementary Table 3: Trimmed and filtered FASTQ QC metrics for 73 samples sequenced using R9.4.1 flow cell.

Supplementary Table 4: Raw FASTQ QC metrics for 29 samples sequenced on the R10.4.1 flow cell.

S.No.	Sample	Total bases_fastq	Number of reads_fastq	Median read quality_fastq	Median read length_fastq	Read length N50_fastq
1	Family_1_Father	103,239,559,381	19,072,681	18.3	2,416	12,660
2	Family_1_Mother	94,971,191,088	15,192,865	18.6	2,940	13,934
3	Family_1_Proband	92,589,215,167	12,085,760	18.9	3,931	15,948
4	Family_3_Father	113,123,998,342	21,676,581	18.4	3,161	8,918
5	Family_3_Mother	116,301,918,708	18,701,934	18.7	3,545	11,289
6	Family_3_Proband	87,072,186,608	15,485,693	18.6	3,212	10,229
7	Family_4_Brother	109,965,766,880	23,422,189	18.4	2,573	8,633
8	Family_4_Father	113,228,877,921	36,789,519	17.4	1,588	5,733
9	Family_4_Mother	141,506,552,578	43,825,712	18.2	2,201	4,766
10	Family_4_Proband	132,832,199,693	38,925,450	17.7	1,767	6,444
11	Family_8_Father	118,825,762,491	19,717,271	18.5	2,937	13,068
12	Family_8_Mother	85,999,081,935	17,628,250	18.1	2,169	11,659
13	Family_8_Proband	118,997,710,924	39,550,354	17.5	1,291	6,936
14	Family_8_Sister	108,598,671,107	38,548,894	17.5	1,246	6,342
15	Family_9_Father	85,619,101,476	19,266,224	18	2,115	9,320
16	Family_9_Mother	94,368,142,329	18,562,800	18.3	2,438	10,744
17	Family_9_Proband	97,864,666,827	18,342,286	18.5	2,654	10,793
18	Family_10_Father	70,549,900,680	18,185,887	17.4	1,725	9,269
19	Family_10_Mother	82,888,052,802	18,577,981	17.7	1,829	11,691
20	Family_10_Proband	63,104,207,596	17,216,211	17.3	1,419	10,367
21	Family_13_Father	114,848,402,635	45,816,218	17.5	1,402	4,223
22	Family_13_Mother	114,075,012,024	30,447,203	18.2	2,440	5,827
23	Family_13_Proband	88,848,902,151	14,363,249	18.6	3,485	11,128
24	Family_15_Father	140,253,530,958	53,919,664	18	1,839	3,738
25	Family_15_Mother	97,244,846,577	13,683,618	18.8	4,258	12,029
26	Family_15_Proband	97,994,614,810	13,926,547	18.9	4,267	11,665
27	Family_18_Father	80,516,750,327	11,369,179	18.9	4,031	13,203
28	Family_18_Mother	109,695,720,308	15,472,894	18.8	4,070	13,083
29	Family_18_Proband	98,692,514,416	17,119,029	18.3	2,972	11,625

Metrics generated on raw ONT fastq files using NanoComp. Total bases and read lengths are reported in base pairs. Median read quality is reported as a Phred quality score. Read length N50 represents the read length at which 50% of the total sequenced bases are contained in reads of that length or longer.

Supplementary Table 5: Trimmed and filtered FASTQ QC metrics for 29 samples sequenced on the R10.4.1 flow cell.

S.No.	Sample	Total bases_fastq	Number of reads_fastq	Median read quality_fastq	Median read length_fastq	Read length N50_fastq
1	Family_1_Father_trimmed_filtered	99,244,277,297	13,811,178	20	3,713	13,467
2	Family_1_Mother_trimmed_filtered	92,199,304,222	11,837,330	20	4,057	14,553
3	Family_1_Proband_trimmed_filtered	90,951,538,826	10,353,085	20	4,655	16,362
4	Family_3_Father_trimmed_filtered	109,416,195,762	17,629,893	19.8	4,009	9,186
5	Family_3_Mother_trimmed_filtered	113,396,319,373	15,601,139	19.9	4,400	11,575
6	Family_3_Proband_trimmed_filtered	84,417,228,112	12,475,551	19.9	4,225	10,522
7	Family_4_Brother_trimmed_filtered	105,342,025,398	18,091,953	20	3,500	9,062
8	Family_4_Father_trimmed_filtered	103,577,498,415	23,252,450	19.6	2,660	6,416
9	Family_4_Mother_trimmed_filtered	132,776,903,281	33,955,879	19.9	2,774	4,999
10	Family_4_Proband_trimmed_filtered	123,415,607,156	26,496,179	19.7	2,720	7,075
11	Family_8_Father_trimmed_filtered	115,014,710,388	14,882,185	20	4,292	13,625
12	Family_8_Mother_trimmed_filtered	82,178,628,692	12,482,344	19.9	3,305	12,570
13	Family_8_Proband_trimmed_filtered	106,941,920,650	22,673,733	19.9	2,334	8,367
14	Family_8_Sister_trimmed_filtered	96,594,896,072	21,844,199	19.9	2,230	7,795
15	Family_9_Father_trimmed_filtered	81,317,020,310	13,890,816	19.8	3,131	10,080
16	Family_9_Mother_trimmed_filtered	90,542,553,942	13,817,885	19.9	3,506	11,434
17	Family_9_Proband_trimmed_filtered	94,327,855,305	14,229,215	20	3,601	11,374
18	Family_10_Father_trimmed_filtered	66,223,161,392	12,180,375	19.4	2,664	10,457
19	Family_10_Mother_trimmed_filtered	78,603,953,368	12,672,873	19.6	2,909	12,748
20	Family_10_Proband_trimmed_filtered	58,518,622,374	10,493,016	19.5	2,515	11,748
21	Family_13_Father_trimmed_filtered	101,630,406,215	27,691,586	19.8	2,281	4,903
22	Family_13_Mother_trimmed_filtered	108,131,854,219	23,644,553	19.8	3,148	6,075
23	Family_13_Proband_trimmed_filtered	86,751,020,653	12,365,847	19.7	4,116	11,426
24	Family_15_Father_trimmed_filtered	127,815,639,533	38,703,513	19.9	2,456	3,992
25	Family_15_Mother_trimmed_filtered	95,632,859,475	12,303,919	19.8	4,730	12,221
26	Family_15_Proband_trimmed_filtered	96,397,486,964	12,528,494	19.8	4,722	11,843
27	Family_18_Father_trimmed_filtered	78,969,944,307	9,847,631	20	4,745	13,470
28	Family_18_Mother_trimmed_filtered	107,595,012,823	13,547,749	19.9	4,783	13,314
29	Family_18_Proband_trimmed_filtered	95,595,580,967	13,557,441	19.7	3,991	12,103

Reads shorter than 1,000 bp or with a quality score below 9 were removed using NanoFilt, with an additional 40 bp and 20 bp trimmed from the start and end of each read, respectively. Lambda genome reads were removed using NanoLyse. Metrics generated on trimmed and filtered ONT fastq files using NanoComp.

Supplementary Table 6: Alignment statistics for all 74 samples

Supplementary Table 7: Per-family SV counts by SV type across all 24 SGP LRS families.

S.No.	Family_ID	Total_SVs	INS	DEL	DUP	INV	TRA
1	Family_1	23,024	12,418	10,087	17	37	465
2	Family_2	23,527	12,709	10,317	18	39	444
3	Family_3	23,403	12,550	10,265	24	49	515
4	Family_4	24,276	12,973	10,542	37	45	679
5	Family_5	23,869	12,790	10,425	23	52	579
6	Family_6	23,809	12,898	10,378	16	56	461
7	Family_7	23,930	12,780	10,549	21	46	534
8	Family_8	24,001	12,853	10,471	29	39	609
9	Family_9	23,655	12,717	10,369	27	35	507
10	Family_10	23,633	12,812	10,243	33	42	503
11	Family_11	23,374	12,577	10,370	21	45	361
12	Family_12	24,656	13,248	10,801	21	44	542
13	Family_13	23,499	12,610	10,247	31	44	567
14	Family_14	25,009	13,489	11,080	17	44	379
15	Family_15	23,650	12,663	10,280	29	44	634
16	Family_16	24,012	12,933	10,467	26	57	529
17	Family_17	23,797	12,834	10,535	13	38	377
18	Family_18	23,273	12,577	10,138	23	38	497
19	Family_19	23,863	12,935	10,469	21	44	394
20	Family_20	23,819	12,724	10,608	23	45	419
21	Family_21	23,917	12,811	10,452	33	50	571
22	Family_22	24,001	12,859	10,453	28	52	609
23	Family_23	24,041	12,877	10,527	23	47	567
24	Family_24	23,609	12,709	10,424	25	33	418

SV counts were derived from family-based multi-sample VCF files after quality and depth filtering. SV types reported are insertions (INS), deletions (DEL), duplications (DUP), inversions (INV) and translocations (TRA). Total_SVs represents the sum of all SV types per family.

Supplementary Table 8: Genome-wide *de novo* SVs identified in secondary analysis and their curation outcomes.

Supplementary Table 9: Genome-wide compound heterozygous (SNV+SV) candidates from secondary analysis and their curation outcomes.

Supplementary Table 10: SV annotations for genome-wide *de novo* SVs across all SGP LRS cohort families.

Supplementary Table 11: Genome-wide Compound Heterozygous scenarios (SV+SNV) identified across all families in the SGP LRS cohort.

Supplementary Figures

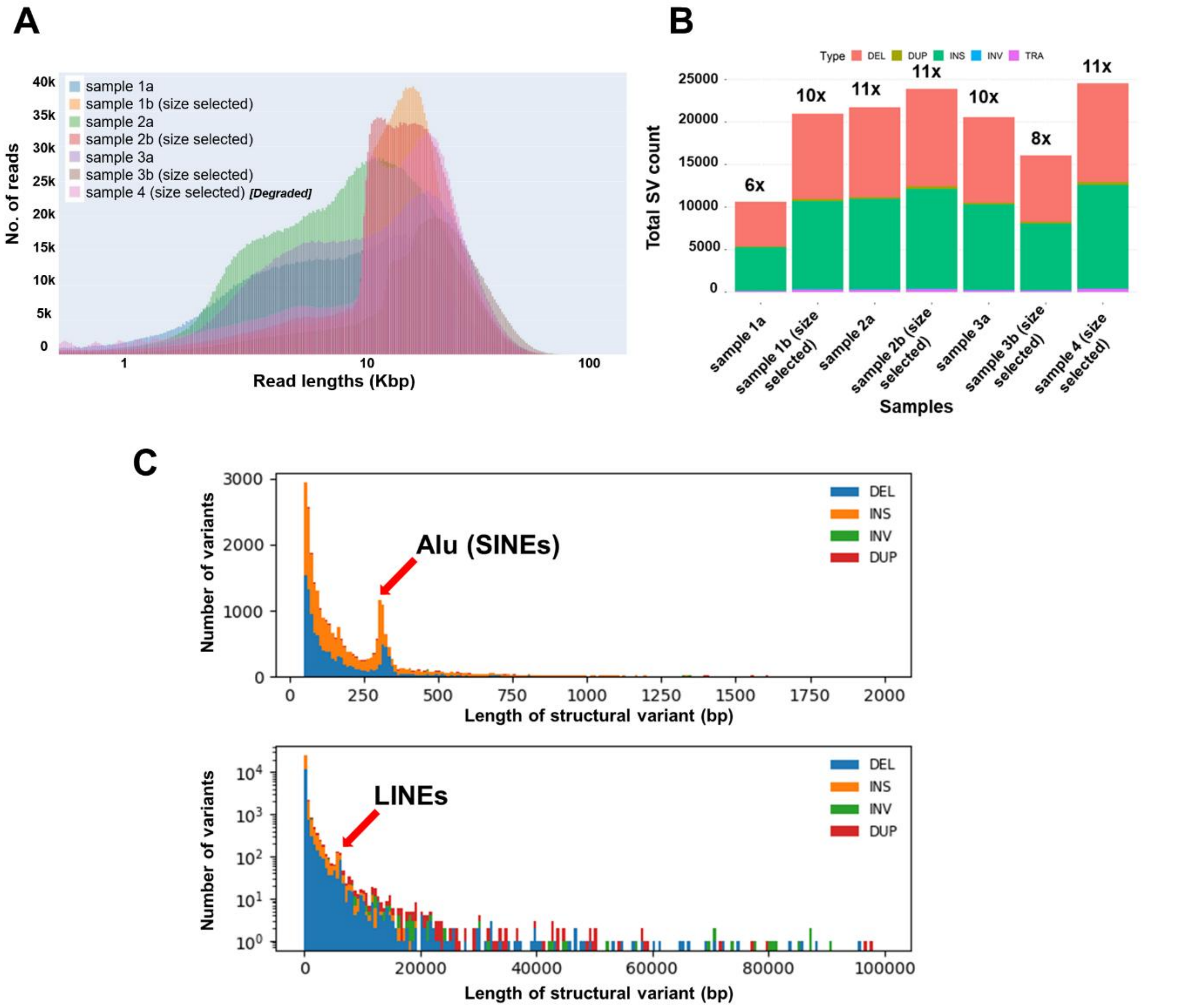

Supplementary Figure 1: (A) Histogram of log-transformed read length (in kb) for size-selected and non-size-selected samples in the pilot study (n=7). (B) Histogram of total SVs discovered in each of the 7 samples. Sample 4 (size-selected) refers to the degraded sample. (C) SV length distribution plots plotted via surpyvor up to 2 kb, binned per 10 bp (upper panel) and up to 100 kb, binned per 500 bp (lower panel). Spikes at ~300 bp and ~6 kb correspond to Alu and LINE-1 elements.

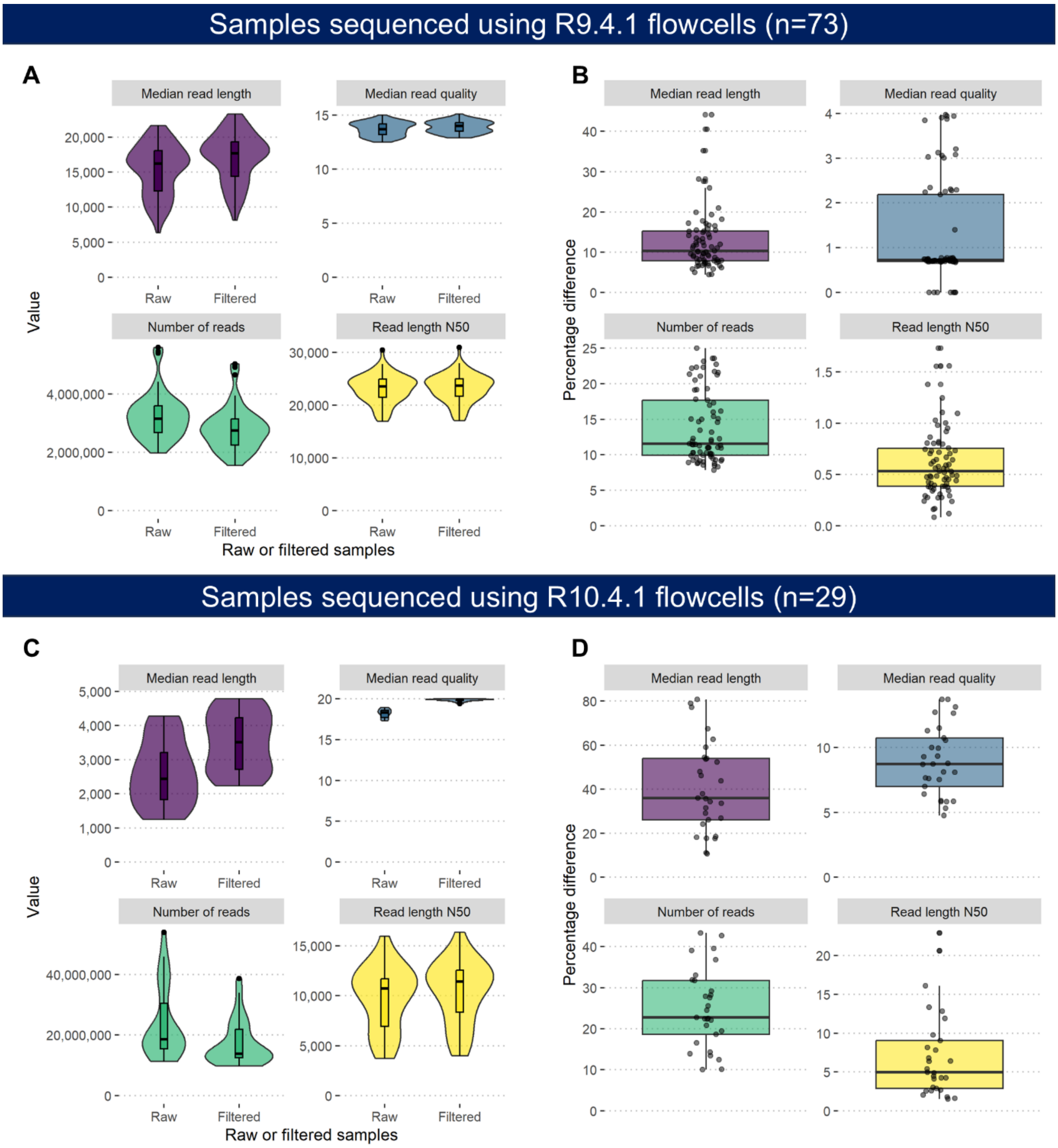

Supplementary Figure 2: Comparison of QC metrics between raw and filtered samples sequenced using R9.4.1 (A, B; n=73) and R10.4.1 (C, D; n=29) flow cells. (A, C) Median read length, median read quality, number of reads, and read length N50 are shown for raw and filtered samples using violin plots with embedded boxplots. (B, D) Percentage differences between raw and filtered values for each QC metric are shown as boxplots with overlaid jittered data points.

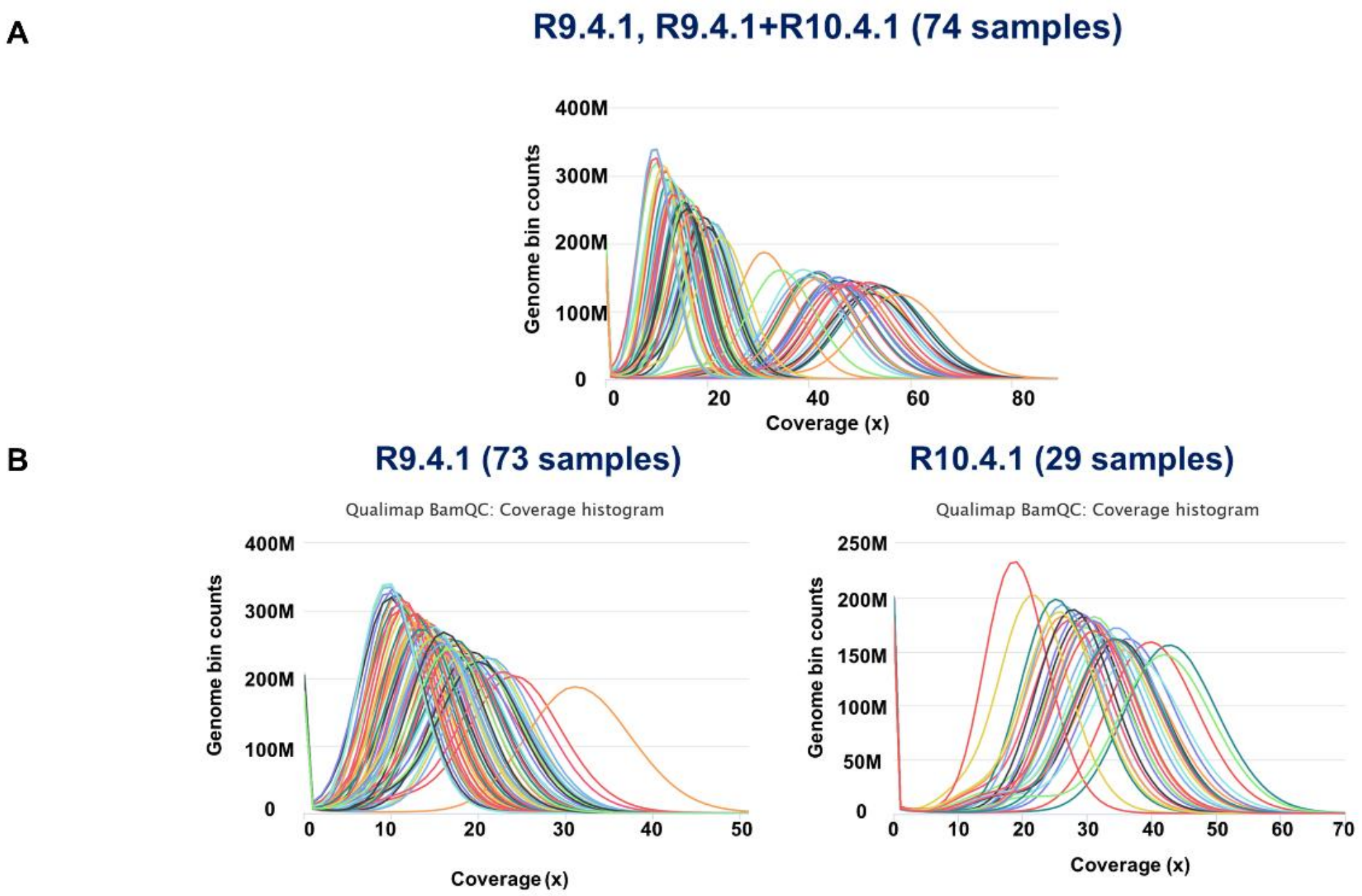

Supplementary Figure 3: A: Coverage histogram for SGP LRS samples (n=74) showing the distribution of the number of locations in the reference genome with a given depth of coverage. Each sample is represented by a different line colour. The average depth of coverage distribution ranges from 10x to 57x across the 74 samples. The plot contains samples sequenced only using R9.4.1 flow cell, merged samples (R9.4.1+R10.4.1), and one sample sequenced only on R10.4.1.

B: Coverage histogram for samples sequenced using only R9.4.1 flow cells (left) vs R10.4.1 flow cells (right) calculated using Qualimap and plotted using MultiQC. Samples on the right show an average coverage ranging from ~19x-45x, whereas samples on the left show an average coverage which ranges from about 10x to 31x.

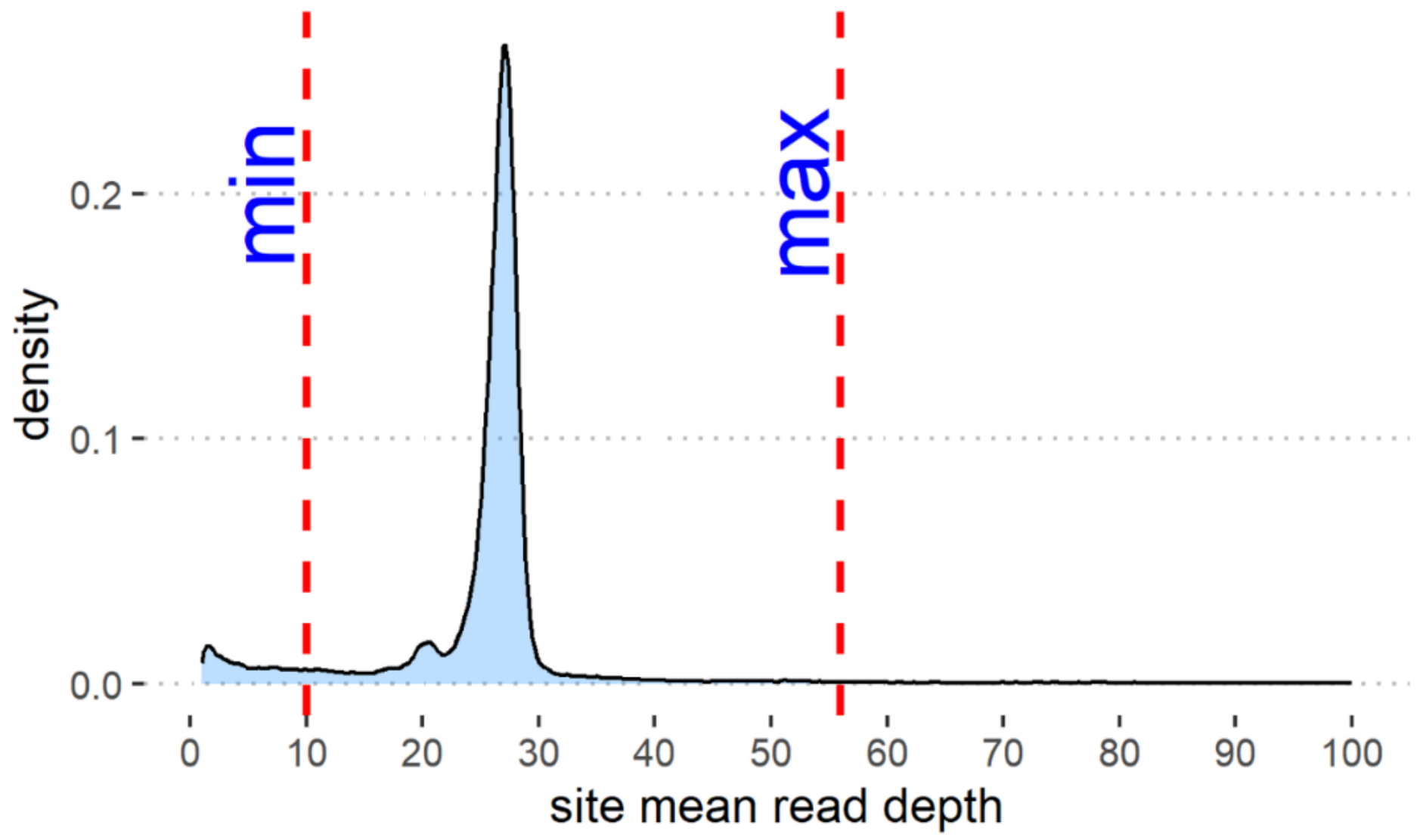

Supplementary Figure 4: Site mean read depth distribution of 74,388 SVs across 74 individuals. Based on this distribution, a depth filter of 10-56x was applied to remove SVs in regions with unusually low or high site mean depth, which are more likely to reflect poor mappability or alignment artefacts in repetitive regions.

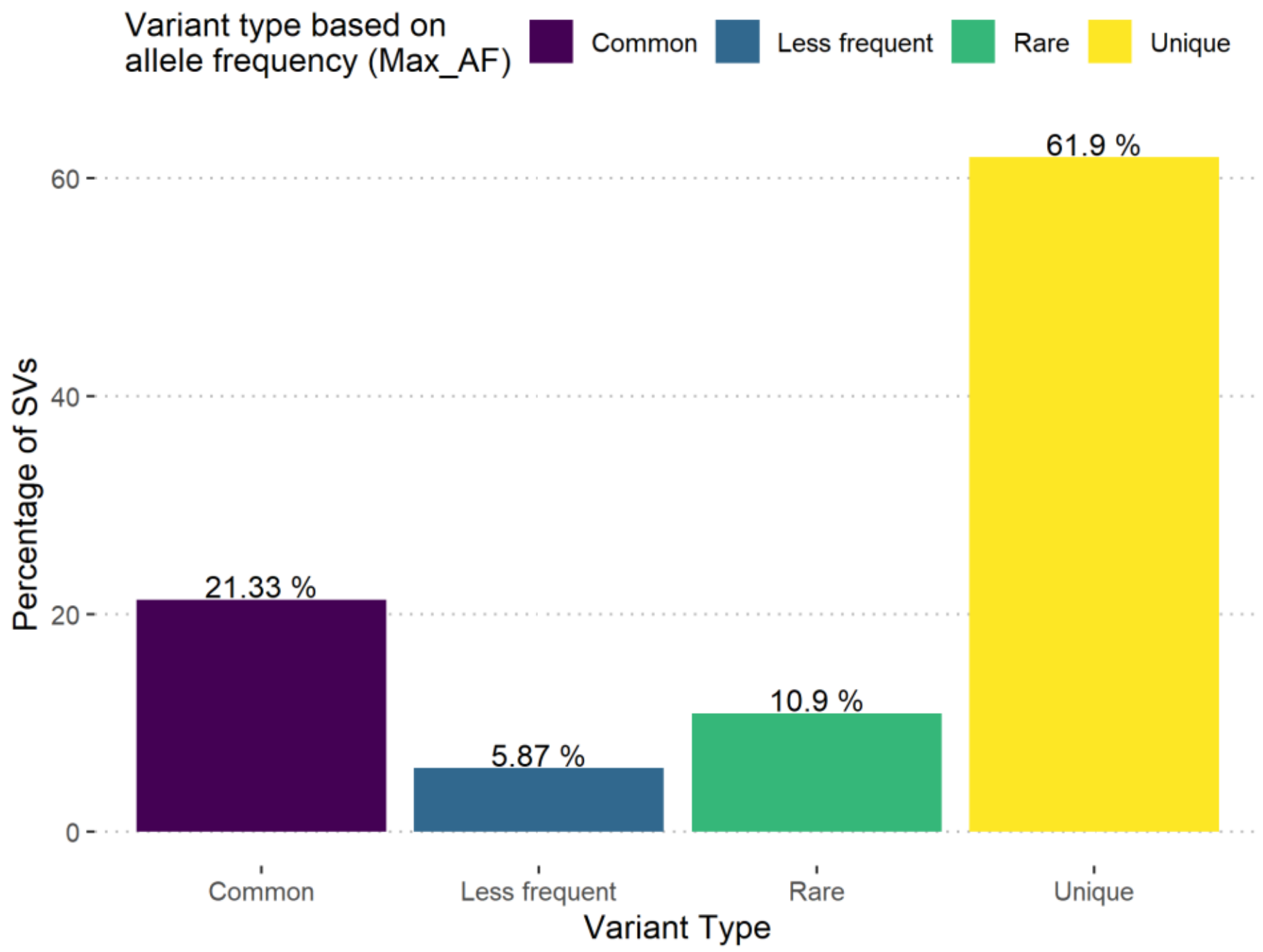

Supplementary Figure 5: Barplot showing percentage of SVs in SGP LRS cohort stratified by variant type based on maximum allele frequency (Max_AF) from SVAFotate.

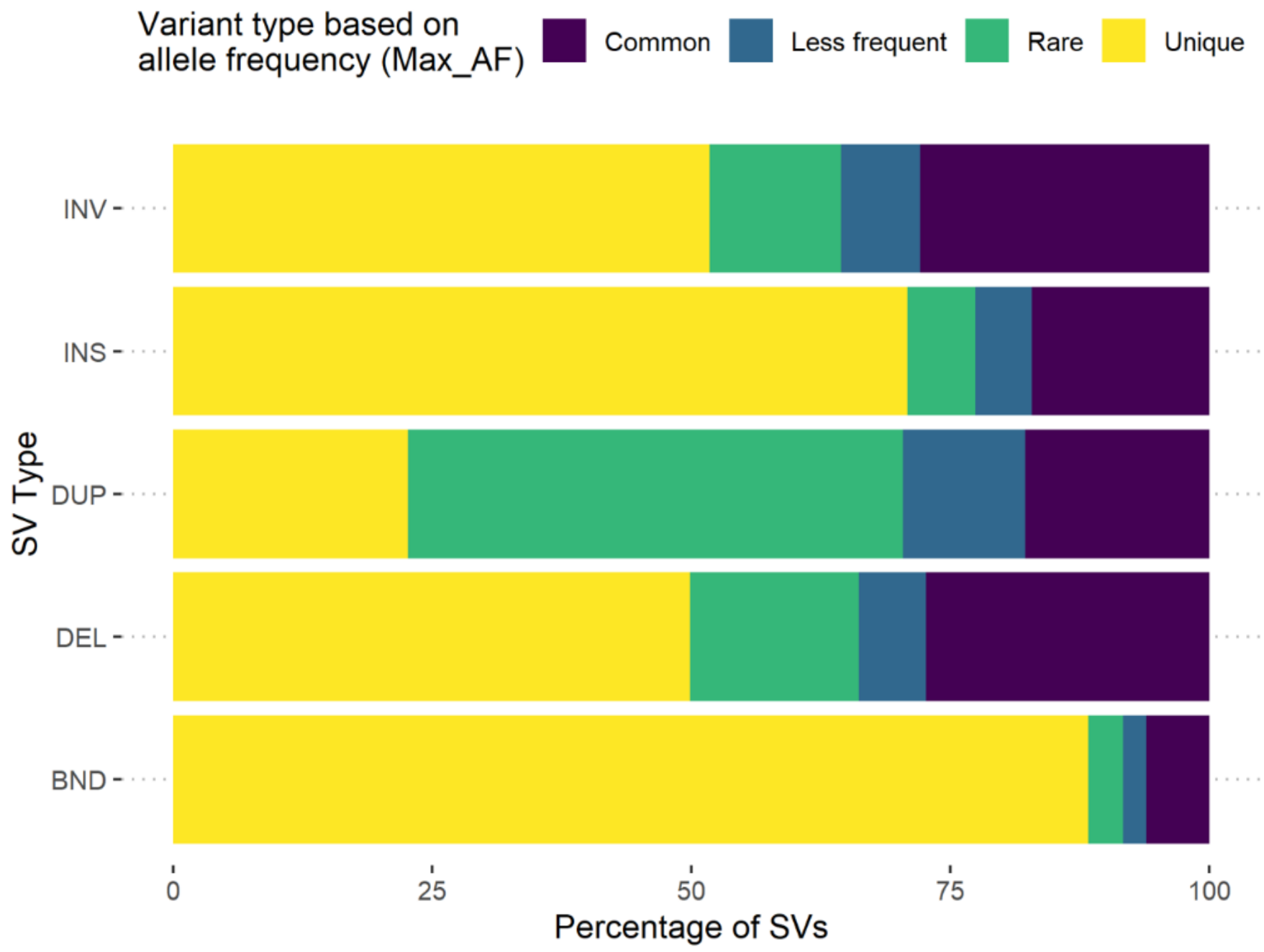

Supplementary Figure 6: Barplot showing the percentages of common, less frequent, rare and unique SVs based on Max_AF stratified by SV type

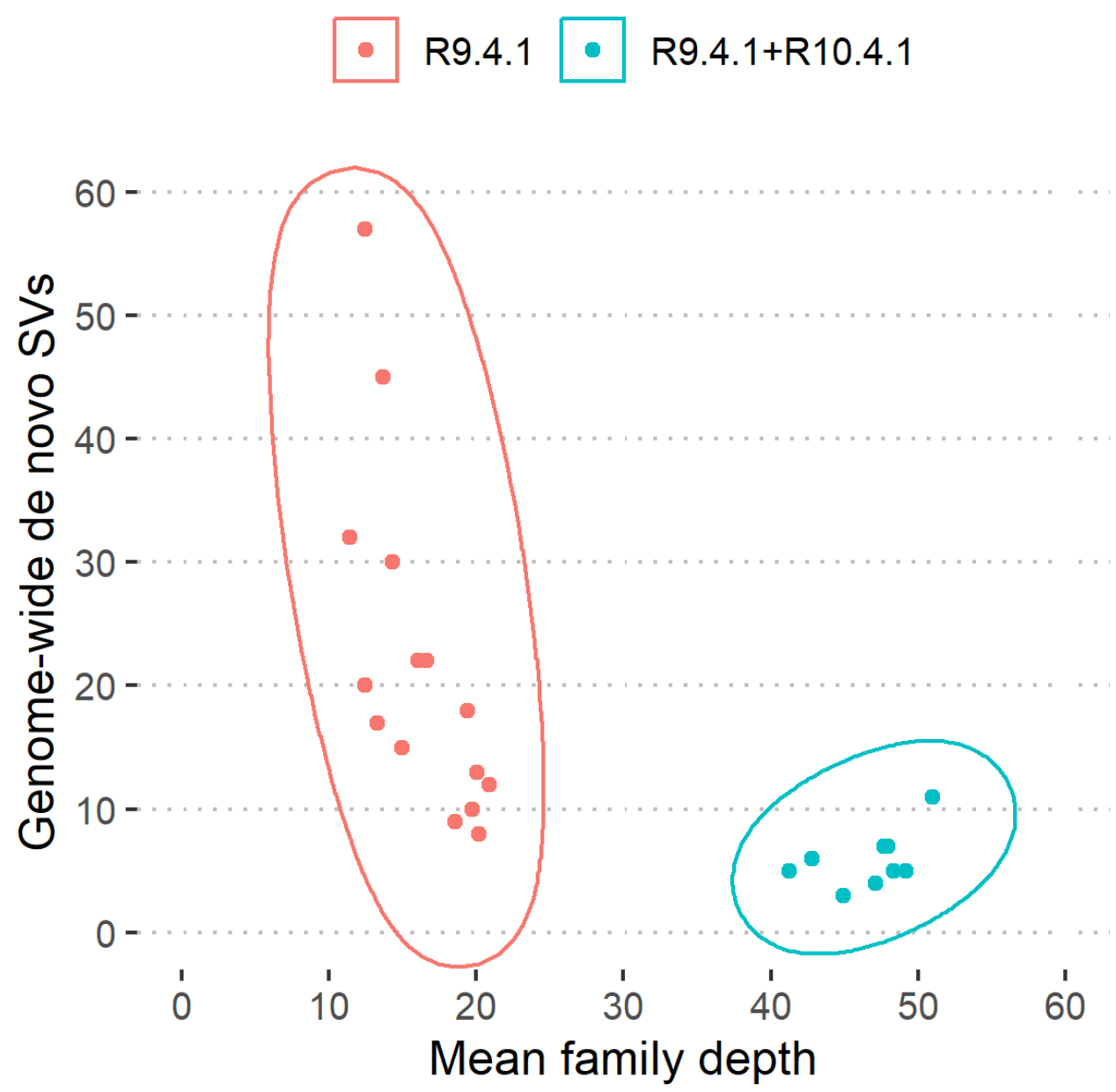

Supplementary Figure 7: Number of genome-wide *de novo* SVs vs. mean family depth. Each dot represents a SGP LRS family, and the different colours indicate whether the samples from that family have been sequenced using only R9.4.1 flow cell (red) or using both R9.4.1 and R10.4.1 flow cells (blue). The two clusters signify that families sequenced using both flow cells led to a higher depth of coverage and a relatively lower number of *de novo* SVs genome-wide than families sequenced using only the R9.4.1 flow cell.

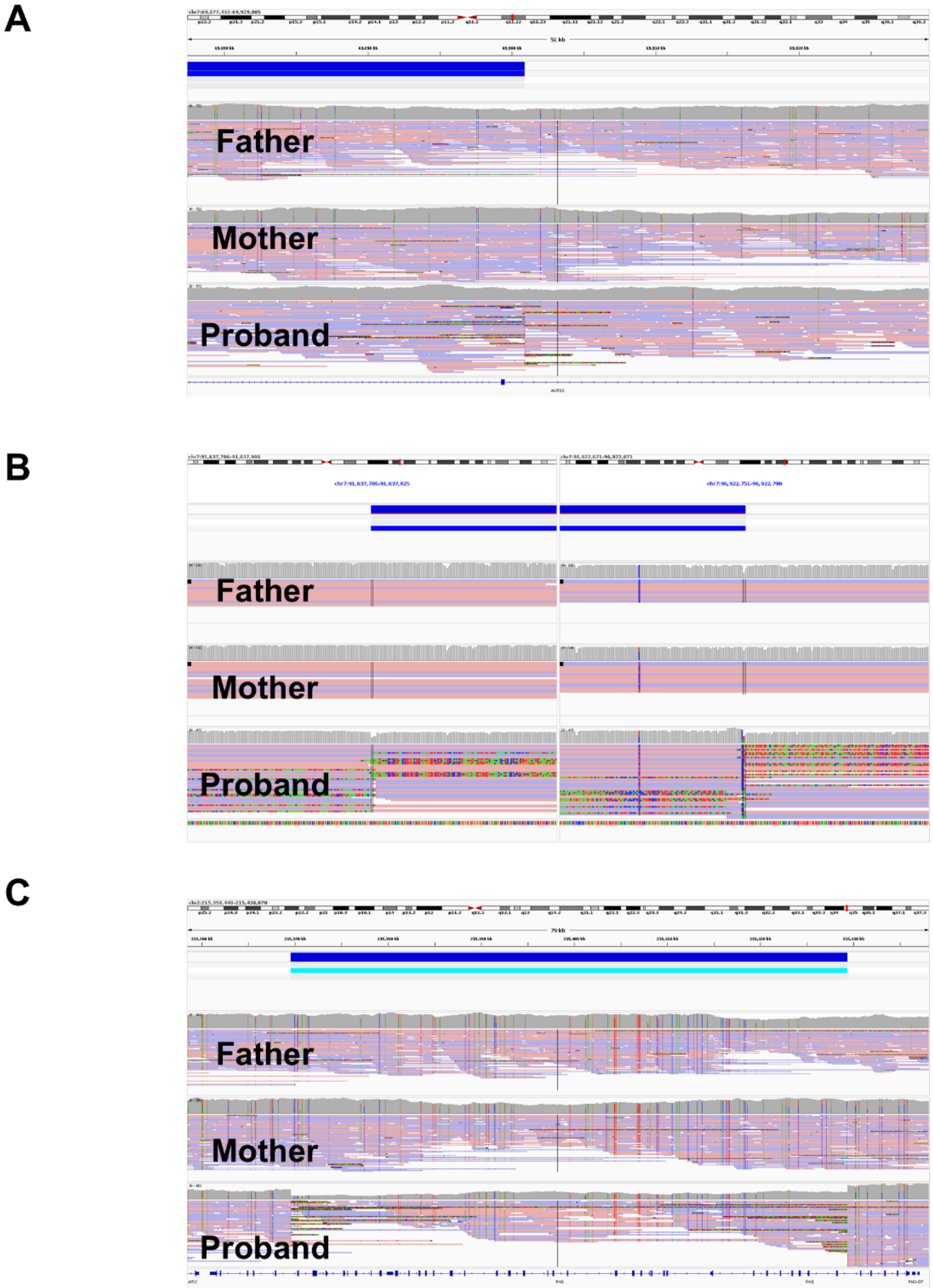

Supplementary Figure 8: SGP LRS IGV plots showing *de novo* SV events in three exemplar families (all plots based on GRCh38 coordinates and sequenced by LRS). (A) One breakpoint of the *de novo* inversion in the proband from exemplar family 1 located in intron 2, disrupting *AUTS2*. (B) *De novo* inversion in the exemplar family 2 proband with breakpoints in two different regions in the non-coding part of chromosome 7. This IGV plot shows data only from the three family members (proband, mother, father) who were sequenced by LRS. (C) Multi-exon *de novo* heterozygous deletion of *FN1* in the proband from exemplar family 3 disrupting exons 6 to 41.

Supplementary Methods

Patient Recruitment and Sample Collection

Recruitment criteria for SGP LRS

Twenty-four families recruited for the SGP LRS study were selected from those that did not receive a genetic diagnosis in the SGP SRS study [1]. The Scottish families recruited for SRS within the SGP presented a rare phenotype that was believed to be genetic in origin. Families had variable structures (singletons, duos, trios, and quads) and had negative results from prior diagnostic testing, including microarray analysis.

To be selected for SGP LRS, families needed to be trios or quads within the SGP SRS study, with a negative report following both primary assessment (Tiers 1 and 2 variants) and secondary assessment (Exomiser [2]/in-depth investigation of Tier 3 variants) [1]. The selected families had a high index of suspicion of monogenic aetiology, either because multiple siblings or family members had the same phenotype or because of syndromic presentation suggestive of monogenic disease. The focus was therefore on families that might benefit the most from diagnosis through LRS analysis. In addition to the 24 families (comprising 74 participants), an additional 4 participants (resulting in 7 libraries) from the same cohort were included as part of a pilot study for sequencing platform optimisation.

DNA preparation

Details of sample collection and DNA extraction were the same as those used for the SGP SRS study [1]. Briefly, DNA was extracted from blood at various Scottish Regional Genetics Laboratories (Aberdeen, Dundee, Edinburgh, and Glasgow). To

be selected for the SGP LRS study, a minimum of 15 µg of genomic DNA, preferably with a DNA integrity number (DIN) value of > 8.0, was required to be available from all family members.

Sample processing and sequencing

Genomic DNA fragmentation and size selection

~7.5 µg of high-molecular-weight (HMW) genomic DNA was sheared into fragments of ~25-40 kb via Megaruptor 3 (Diagenode) according to the Megaruptor instructions at speed 28. A 1x volume of AMPure XP beads was added to the sheared material, and the supernatant was discarded after 30 minutes at room temperature. The beads were washed twice with 80% ethanol, and the DNA was eluted in 60 µl of TE after incubation at 37°C for 1 hour. The fragment size was estimated via pulsed-field capillary electrophoresis via the Femto Pulse system (Agilent Technologies, Santa Clara, CA, USA). To remove short DNA fragments, 30 µl of sheared DNA was loaded into a well of a BluePippin (Sage Science, Beverly, MA, USA) agarose gel cassette (0.75% DF) with 10 µl of loading solution. After 6 hours, 20-40 kb DNA fragments were collected, following the high-pass DNA size selection instructions using marker S1. A total of 40 µl of eluate was transferred to a fresh tube, and 1x volume AMPure XP beads was added. The beads were incubated for 30 minutes at room temperature, and two washes with 80% ethanol were performed. The DNA was eluted in 50 µl of nuclease-free water after 1 hour of incubation at 37°C. DNA was quantified via the Qubit dsDNA High Sensitivity Assay (Thermo Scientific, Wilmington, NC, USA).

Library preparation for Oxford Nanopore genome sequencing

For the pilot study, four participants were selected from the SGP SRS study. For the first three participants, two libraries were constructed, where shearing and size selection were not performed in the first library, whereas shearing and size selection were performed in the second library. Shearing and size selection resulted in the concentration of DNA fragments with lengths between 10 and 40 kb. A sample of partially degraded genomic DNA was available from the fourth selected participant, with a low proportion of high-molecular-weight DNA (less than 40% of the gDNA was >40 kb). Size selection was also performed on this sample, with no further shearing. This resulted in a total of 7 libraries being sequenced on the ONT PromethION Beta machine for the pilot study. The same library preparation and sequencing method was used for family-based samples (n=74).

For the substantive family study, 73 samples were sequenced on ONT R9.4.1 flow cells via a V9 ligation sequencing kit (SQK-LSK109, Oxford Nanopore Technologies, UK). This included the four participants from the pilot study, who were sequenced as part of the expanded cohort. A further 28 of these samples with adequate residual DNA were sequenced via the R10.4.1 (FLO-PRO114M) flow cell via the V14 ligation sequencing kit (SQK-LSK114, Oxford Nanopore Technologies, UK) for library preparation. This was done to achieve greater depth of coverage by utilising the higher yield of the R10.4.1 ONT flow cells. One additional sample was sequenced only on an R10.4.1 flow cell, resulting in a total of 74 samples sequenced for the family-based SV study (Additional File 1: Supplementary Table 1). No size selection was performed on samples sequenced on the R10.4.1 flow cells.

~1-2 µg of sheared and size-selected DNA in a volume of 47 µl of nuclease-free water was used with the SQK-LSK109 or SQK-LSK114 ligation sequencing kits (Oxford Nanopore Technologies, UK), depending on the flow cell type used, for

library preparation following Oxford Nanopore recommendations. Individual sample preparations were loaded onto a single R9.4.1 and run on a PromethION Beta (Oxford Nanopore Technologies, UK) for 72 hours or a single R10.4.1 flow cell and run on the PromethION 24 (Oxford Nanopore Technologies, UK) for 90 hours. Sequencing was carried out at the Edinburgh Genomics facility, University of Edinburgh, UK.

SV analysis

Software benchmarking

To identify an optimal pipeline for SV detection using ONT LRS data, several aligner and SV caller combinations were benchmarked. Single-sample benchmarking was performed using Oxford Nanopore sequencing data from the Ashkenazi Jewish trio son (Genome in a bottle [3] or GIAB HG002) with three long-read aligners (minimap2 [4] v2.23-r1111, NGMLR [5] v0.2.7 and lra [6] v1.3.2) and two SV callers (cuteSV [7] v1.0.13 and Sniffles v2.0.2 [8]) at ~33x coverage. Performance was assessed using precision, recall and F1 score calculated using truvari v3.1.0 [9] *bench* program.

The minimap2-cuteSV combination showed the best overall performance for single-sample SV detection, achieving the highest F1 score (0.93) with balanced precision and recall. At lower coverage (~10x), performance decreased across all methods, but minimap2-cuteSV remained the best-performing combination. These trends were consistent when restricting to correctly genotyped SVs.

For family-based analysis, cuteSV and Sniffles2 were compared using force-calling and multi-sample approaches to assess Mendelian consistency and genotype completeness in one SGP LRS trio. Sniffles2 multi-sample calling showed lower genotype missingness and a higher proportion of consistent genotypes compared

with force-calling approaches. Based on this benchmarking, minimap2-Sniffles2 was selected for multi-sample SV calling.

SV analysis and filtering

In the pilot study (n=7), base calling and sample demultiplexing were performed via Guppy (Oxford Nanopore Technologies, UK) v4.0.11+f1071ce. The resulting reads that passed Guppy QC were put into a FASTQ file with a “pass” tag. “pass” tagged reads were processed further where reads <500 bp, read quality score <9, were discarded and 40 bp and 20 bp were chopped off from start and end of the reads respectively using NanoFilt v2.8.0. Lambda genome reads were removed using NanoLyse v1.2.0. FASTQ statistics for the 7 samples were generated via NanoComp v1.17.0.

The alignment of trimmed and filtered FASTQ files was performed via minimap2 v2.23-r1111 against the GRCh38 build of the human reference genome via the parameters -x map-ont and –MD. SVs were called via cuteSV v1.0.13. BAM file statistics were calculated via Qualimap [10] *bamqc* v2.2.2-dev and NanoComp [11] v1.17.0.

In the family-based study (n=74), all samples were sequenced via the R9.4.1 flow cell on the PromethION Beta, and 29 additional samples were sequenced via the R10.4.1 flow cells on the PromethION 24 machine (Additional File 1: Supplementary Table 1). For base calling and demultiplexing, Guppy v5.1.13+b292f4d was used in the PromethION Beta machine, whereas Guppy v6.5.7+ca6d6af was used in the PromethION 24 machine. “pass” tagged reads were processed further where reads <1000 bp and reads with quality scores <9 were discarded and 40 bp and 20 bp were chopped off from start and end of the reads respectively using NanoFilt v2.8.0.

Lambda genome reads were removed using NanoLyse v1.2.0. Read statistics were calculated in the same way as above.

Trimmed and filtered reads (FASTQ) were aligned to the GRCh38 reference genome via minimap2 v2.23-r1111 with parameters -x map-ont, --MD and --secondary=no and coordinate sorted and indexed via samtools [12, 13] v1.15.1. The FASTQ files generated from each flow cell type were processed separately to produce trimmed and filtered reads. For samples that were sequenced on both R9.4.1 and R10.4.1 flow cells, BAM files were merged into a single combined BAM file. QC statistics were calculated on the resulting BAM files via NanoComp v1.17.0, Qualimap *bamqc* v2.2.2 and samtools *flagstat* v1.15.1. Multi-sample SV calling was performed across all SGP LRS samples (n=74) via Sniffles v2.0.6, where first, SNF (Sniffles2 file, a specialised binary file format introduced in [8]) files were produced from the BAM files via --long-del-length 1000000, --long-ins-length 1000000, --long-dup-length 1000000, --minsvlen 50 and --tandem-repeats, and then multi-sample SV calling was performed using all 74 SNF files, producing a fully genotyped multi-sample VCF file. SVs overlapping with genome gaps and contamination/false duplication were removed. Information on gaps in regions such as short_arm, heterochromatin, telomere, contig and scaffold regions was downloaded from UCSC for hg38, and any overlapping SVs were removed. Monomorphic loci and any loci with a >90% missing data rate were also removed. Those SVs that had average site mean depth (or mean depth per site averaged across all individuals) < 10 and > 56 were removed. This filtering removed SVs in low-coverage regions and those with inflated depth, likely reflecting poor mappability or alignment artefacts, particularly in repetitive regions. The upper threshold (56x) was derived empirically from the observed distribution, corresponding to approximately twice the mean site-level depth. SVs with a QUAL <

25 were removed. Furthermore, SVs with lengths of 50 bp or more and missing lengths (to account for translocations) were retained. All filtering was performed via BCFtools v1.15.1 and bedtools v2.30.0. Finally, SV annotation and functional impact prediction were performed via VEP [14] v110.1. ClinVar SV annotations and OMIM phenotype annotations were also added. Panel gene-specific SVs were selected via a VEP *filter* in a custom script. SV allele frequency annotation was performed via SVAFotate [15] v0.1.0 (parameters: -f 0.5, --cov 0, --lim, 1000000, -a mis), which annotates SVs via population-level allele frequency (AF) values from four major databases: CCDG [16], gnomAD-SV [17] (v4.2.1), 1000G [18] and TopMed SVs [19]. SVAFotate annotates each SV with the maximum AF (Max_AF) from all matching SVs across all four databases. In this study, SVs are denoted as common if Max_AF >= 0.05, low frequency if Max_AF < 0.05 and Max_AF >= 0.01 and rare if Max_AF < 0.01. If Max_AF = 0, then the SV is unique to the SGP LRS cohort. The unique SV might have a different SV type in the population databases. The complete SV analysis and filtering schematic diagram is shown in Figure 1.

SV prioritisation

To prioritise *de novo* SVs, the multi-sample VCF file with autosomal SVs was processed via BCFtools v1.15.1 to obtain family-specific VCF files (n=24), after which monomorphic loci were removed. Slivar [20] v0.3.1 was used to find *de novo* SVs in the family VCFs, which followed this rule: the proband's genotype is a heterozygous alternate or homozygous alternate, the mother and father have a homozygous reference genotype (0/0 for mother and father and 0/1 or 1/1 for proband), the proband, mother and father should have a minimum depth of 5 reads, and there should be no reads in the mother and father supporting the variant/alternate allele, Max_AF < 0.001 and SGP LRS cohort AF < 0.01.

Additionally, SGP LRS cohort AF < 0.015 filtering was also performed separately (keeping all other filtering parameters the same) to detect *de novo* SVs that could have been genotyped incorrectly.

Patient-specific clinician-assigned gene panels were downloaded from PanelApp [21]. The latest available PanelApp diagnostic-grade “green” gene panels at the time of analysis were used. Only those *de novo* SVs that overlapped with genes from panels assigned to the family by their respective clinicians were retained. Only panel genes that showed a monoallelic mode of inheritance (MOI) linked to the disorder represented were retained via a custom script.

To identify SV overlapping genes with homozygous autosomal recessive MOIs for the condition in question, genes annotated with biallelic MOIs were selected from panels associated with the proband. Those SVs were prioritised via slivar where a proband showed a homozygous genotype for the alternate allele and where the mother and father were heterozygous for the alternate allele (0/1 for the mother and father and 1/1 for the proband), all three samples had a depth of at least 5 reads and Max_AF < 0.01 and the SGP LRS cohort AF < 0.05.

Potential compound heterozygotes with an SV in-*trans* with an SNV or small indel were also investigated. Previously generated Illumina short-read data [1] were used to detect SNVs and small indels because of their greater depth, whereas SVs detected from LRS were used for this analysis. Short-read Illumina VCF files with small variants (SNVs and indels) created by Edinburgh Genomics were intersected with long-read structural variant (SV) VCFs. On a per-family basis, proband SVs were retained where they passed all quality filters, the SGP LRS cohort AF was < 0.01, the biotype was "protein_coding", IMPACT was HIGH or MODERATE, and the

overlapping transcript was canonical. The corresponding SNV VCF files were then generated via this subset of regions that passed all the quality filters. The SNV VCFs were then normalised via BCFtools v1.20 to decompose multiallelic sites and then annotated with VEP v110, including annotations for precomputed ‘masked’ spliceAI [22] scores, CADD [23] scores and gnomAD [24] v4.1 allele frequency annotations. SNVs were filtered using gnomAD AF (exomes + genomes) < 0.01, and the IMPACT was either HIGH or MODERATE. A compound heterozygote was considered when at least one SV and SNV passed this filtering with an inheritance pattern suggesting that the variants were in-*trans*. Panel-specific compound heterozygous scenarios were then obtained as a subset of the genome-wide scenarios, focusing on biallelic panel genes.

For X-linked analysis, those families that had an affected male proband with a hemizygous SV and an unaffected/carrier mother with a heterozygous SV were examined; Max_AF < 0.01, the SGP LRS cohort AF < 0.05, and all samples of the family had a depth of at least 5 reads. Both panel-based and chromosome-wide analyses were carried out.

In addition to this primary panel-based approach, a secondary analysis was undertaken to explore variants beyond the predefined gene panels. Genome-wide *de novo* SVs annotated by VEP [14] as HIGH or MODERATE impact were identified from family-specific VCFs containing rare *de novo* SVs using a custom script. These variants were subsequently reviewed in IGV for manual inspection and downstream interpretation.

For genome-wide compound heterozygous analysis, SVs across all OMIM-annotated biallelic disease genes (downloaded from OMIM) were intersected with SNVs for

each proband. SNVs were filtered to retain variants with HIGH or MODERATE VEP impact, PASS quality filters, and a gnomAD v4.1 joint allele frequency (exomes + genomes) < 0.01. SVs from both *de novo* and inherited SV VCF files were included in this analysis. VEP impact was taken with respect to the canonical transcript. Candidate compound heterozygous combinations were identified where an SV and SNV affected the same gene and were consistent with an *in trans* configuration.

SRS SV analysis using SVRare

Genome sequencing and data processing in the 100,000 Genomes Project have been described elsewhere [25]. In brief, libraries were prepared from DNA extracted from blood via the TruSeq PCR-Free High-Throughput Kit, and 150 bp paired-end reads were sequenced in a single lane of an Illumina HiSeqX instrument. The reads were aligned to the GRCh38 build or GRCh37 build of the human reference genome via the iSAAC Aligner [26], and the SVs were called via Manta [27] and Canvas [28].

SVRare [29] was used to collate the deletions, duplications and inversions of the SVs into a database and annotate each SV with an estimated recurrence frequency, the potential to disrupt the protein-coding sequence of the overlapping genes, and its relevance to the patient's disease or phenotypes. SVs with GRCh37 coordinates were lifted over to GRCh38, and an 80% overlap threshold was used for recurrence frequency estimation.

References

1. Hocking LJ, Andrews C, Armstrong C, Ansari M, Baty D, Berg J, et al. Genome sequencing with gene panel-based analysis for rare inherited conditions in a publicly funded healthcare system: implications for future testing. European Journal of Human Genetics. 2022.
2. Smedley D, Jacobsen JO, Jager M, Kohler S, Holtgrewe M, Schubach M, et al. Next-generation diagnostics and disease-gene discovery with the Exomiser. Nat Protoc. 2015;10(12):2004-15.
3. Zook JM, Catoe D, McDaniel J, Vang L, Spies N, Sidow A, et al. Extensive sequencing of seven human genomes to characterize benchmark reference materials. Sci Data. 2016;3:160025.
4. Li H. Minimap2: pairwise alignment for nucleotide sequences. Bioinformatics. 2018;34(18):3094-100.
5. Sedlazeck FJ, Rescheneder P, Smolka M, Fang H, Nattestad M, von Haeseler A, et al. Accurate detection of complex structural variations using single-molecule sequencing. Nat Methods. 2018;15(6):461-8.
6. Ren J, Chaisson MJP. lra: A long read aligner for sequences and contigs. PLoS Comput Biol. 2021;17(6):e1009078.
7. Jiang T, Liu Y, Jiang Y, Li J, Gao Y, Cui Z, et al. Long-read-based human genomic structural variation detection with cuteSV. Genome Biol. 2020;21(1):189.
8. Smolka M, Paulin LF, Grochowski CM, Horner DW, Mahmoud M, Behera S, et al. Detection of mosaic and population-level structural variants with Sniffles2. Nature Biotechnology. 2024;42(10):1571-80.
9. English AC, Menon VK, Gibbs R, Metcalf GA, Sedlazeck FJ. Truvari: Refined Structural Variant Comparison Preserves Allelic Diversity. bioRxiv. 2022:2022.02.21.481353.
10. Okonechnikov K, Conesa A, Garcia-Alcalde F. Qualimap 2: advanced multi-sample quality control for high-throughput sequencing data. Bioinformatics. 2016;32(2):292-4.
11. De Coster W, Rademakers R. NanoPack2: population-scale evaluation of long-read sequencing data. Bioinformatics. 2023;39(5).
12. Li H, Handsaker B, Wysoker A, Fennell T, Ruan J, Homer N, et al. The Sequence Alignment/Map format and SAMtools. Bioinformatics. 2009;25(16):2078-9.
13. Danecek P, Bonfield JK, Liddle J, Marshall J, Ohan V, Pollard MO, et al. Twelve years of SAMtools and BCFtools. Gigascience. 2021;10(2).
14. McLaren W, Gil L, Hunt SE, Riat HS, Ritchie GR, Thormann A, et al. The Ensembl Variant Effect Predictor. Genome Biol. 2016;17(1):122.
15. Nicholas TJ, Cormier MJ, Quinlan AR. Annotation of structural variants with reported allele frequencies and related metrics from multiple datasets using SVAFotate. BMC Bioinformatics. 2022;23(1):490.
16. Abel HJ, Larson DE, Regier AA, Chiang C, Das I, Kanchi KL, et al. Mapping and characterization of structural variation in 17,795 human genomes. Nature. 2020;583(7814):83-9.
17. Collins RL, Brand H, Karczewski KJ, Zhao X, Alfoldi J, Francioli LC, et al. A structural variation reference for medical and population genetics. Nature. 2020;581(7809):444-51.
18. Byrska-Bishop M, Evani US, Zhao X, Basile AO, Abel HJ, Regier AA, et al. High-coverage whole-genome sequencing of the expanded 1000 Genomes Project cohort including 602 trios. Cell. 2022;185(18):3426-40 e19.
19. Jun G, English AC, Metcalf GA, Yang J, Chaisson MJ, Pankratz N, et al. Structural variation across 138,134 samples in the TOPMed consortium. Res Sq. 2023.
20. Pedersen BS, Brown JM, Dashnow H, Wallace AD, Velinder M, Tristani-Firouzi M, et al. Effective variant filtering and expected candidate variant yield in studies of rare human disease. NPJ Genom Med. 2021;6(1):60.

21. Martin AR, Williams E, Foulger RE, Leigh S, Daugherty LC, Niblock O, et al. PanelApp crowdsources expert knowledge to establish consensus diagnostic gene panels. Nat Genet. 2019;51(11):1560-5.
22. Jaganathan K, Kyriazopoulou Panagiotopoulou S, McRae JF, Darbandi SF, Knowles D, Li YI, et al. Predicting Splicing from Primary Sequence with Deep Learning. Cell. 2019;176(3):535-48 e24.
23. Rentzsch P, Witten D, Cooper GM, Shendure J, Kircher M. CADD: predicting the deleteriousness of variants throughout the human genome. Nucleic Acids Res. 2019;47(D1):D886-D94.
24. Chen S, Francioli LC, Goodrich JK, Collins RL, Kanai M, Wang Q, et al. A genomic mutational constraint map using variation in 76,156 human genomes. Nature. 2024;625(7993):92-100.
25. Smedley D, Investigators GPP, Smith KR, Martin A, Thomas EA, McDonagh EM, et al. 100,000 Genomes Pilot on Rare-Disease Diagnosis in Health Care - Preliminary Report. N Engl J Med. 2021;385(20):1868-80.
26. Raczy C, Petrovski R, Saunders CT, Chorny I, Kruglyak S, Margulies EH, et al. Isaac: ultra-fast whole-genome secondary analysis on Illumina sequencing platforms. Bioinformatics. 2013;29(16):2041-3.
27. Chen X, Schulz-Trieglaff O, Shaw R, Barnes B, Schlesinger F, Kallberg M, et al. Manta: rapid detection of structural variants and indels for germline and cancer sequencing applications. Bioinformatics. 2016;32(8):1220-2.
28. Roller E, Ivakhno S, Lee S, Royce T, Tanner S. Canvas: versatile and scalable detection of copy number variants. Bioinformatics. 2016;32(15):2375-7.
29. Yu J, Szabo A, Pagnamenta AT, Shalaby A, Giacopuzzi E, Taylor J, et al. SVRare: discovering disease-causing structural variants in the 100K Genomes Project. medRxiv. 2022:2021.10.15.21265069.
